# Recognition by host nuclear transport proteins drives disorder-to-order transition in Hendra virus V

**DOI:** 10.1038/s41598-017-18742-8

**Published:** 2018-01-10

**Authors:** Sarah C. Atkinson, Michelle D. Audsley, Kim G. Lieu, Glenn A. Marsh, David R. Thomas, Steven M. Heaton, Jason J. Paxman, Kylie M. Wagstaff, Ashley M. Buckle, Gregory W. Moseley, David A. Jans, Natalie A. Borg

**Affiliations:** 1 Infection & Immunity Program, Monash Biomedicine Discovery Institute and Department of Biochemistry and Molecular Biology, Monash University, Clayton, Victoria Australia; 2 CSIRO Livestock Industries, Australian Animal Health Laboratory, Victoria, Australia; 3 La Trobe Institute for Molecular Sciences and Department of Biochemistry and Genetics, La Trobe University, Melbourne, Victoria Australia

## Abstract

Hendra virus (HeV) is a paramyxovirus that causes lethal disease in humans, for which no vaccine or antiviral agent is available. HeV V protein is central to pathogenesis through its ability to interact with cytoplasmic host proteins, playing key antiviral roles. Here we use immunoprecipitation, siRNA knockdown and confocal laser scanning microscopy to show that HeV V shuttles to and from the nucleus through specific host nuclear transporters. Spectroscopic and small angle X-ray scattering studies reveal HeV V undergoes a disorder-to-order transition upon binding to either importin α/β1 or exportin-1/Ran-GTP, dependent on the V N-terminus. Importantly, we show that specific inhibitors of nuclear transport prevent interaction with host transporters, and reduce HeV infection. These findings emphasize the critical role of host-virus interactions in HeV infection, and potential use of compounds targeting nuclear transport, such as the FDA-approved agent ivermectin, as anti-HeV agents.

## Introduction

Hendra virus (HeV) is a *Henipavirus* belonging to the *Paramyxoviridae* family of single-stranded negative-sense RNA viruses, closely related to Nipah virus (NiV)^1^. Although the primary natural reservoir of HeV is flying foxes of the genus *Pteropus*, all recorded human infections have been transmitted through contact with infected horses, where severe respiratory and/or neurological disease results in a high incidence of lethality (~57%)^2^. While HeV outbreaks have only been recorded in Australia, anti-HeV antibody responses have been discovered in *Pteropus* and/or *Eidolon* species on the South East and West coast of Africa^3–5^. Despite the potential for the geographical spread of HeV, and the high fatality rate in humans, there is currently no antiviral agent available to treat HeV infection.

The potent pathogenicity of *Henipavirus* is in part due to its ability to suppress host type I interferon (IFN-I) responses via the products of the polycistronic P gene. The unedited *Henipavirus* P gene transcript generates not only the phosphoprotein (P), but also, mRNAs that encode the partly frame-shifted V and W proteins, which share an identical N-terminal region (residues 1–405) with P, but have unique C-terminal regions. Although the HeV V C-terminal region (residues 406–457) is proposed to adopt a novel zinc finger fold as per the highly conserved parainfluenza virus 5 (PIV5)^6^ and the N-terminal 50 residues are predicted to be alpha (α)-helical, the HeV shared P/V/W residues 51–405 are intrinsically disordered and lack persistent structure in solution^7^. The plasticity of intrinsically disordered proteins (IDPs) or regions (IDRs) enables them to bind multiple partners, conferring functional versatility^8^. HeV V binds to numerous cytosolic host proteins to limit or prevent IFN-I induction, including the double-stranded RNA sensor MDA5 (melanoma differentiation-associated protein 5)^9,10^, and the signal transducer and activator of transcription (STAT) proteins 1 and 2^11^. LGP2 (laboratory of genetics and physiology 2)^12^ and PLK1 (polo-like kinase)^13^ also appear to be HeV V interacting partners; both proteins regulate MDA5-dependent IFN-I induction, although the consequence of their interaction with V is unknown^10,11,13,14^.

Although paramyxoviruses replicate entirely in the cytoplasm of the host cell, a number of paramyxovirus proteins have been detected in the nucleus, including matrix (M) and W (a P-gene transcript) from HeV and/or the closely related NiV^15,16^. Importantly, this allows access to host transcription processes. All nuclear translocation across the nuclear envelope occurs via membrane-embedded nuclear pore complexes (NPCs). Proteins with a Stokes radius greater than 2.6 nm (~40 kDa globular proteins) require signal-dependent nuclear transport and specific trafficking receptors^17^. The best-characterized nuclear import pathway is that mediated by the importin α1/β1 which recognizes cargo proteins bearing a specific nuclear localization signal (NLS); this pathway is known to be inhibited by the importin α-targeting compound ivermectin^18^. The best-characterized nuclear export pathway is that mediated by the importin superfamily member exportin-1, which recognizes cargo proteins bearing leucine-rich nuclear export signals (NESs) and can be specifically inhibited by the compound leptomycin B (LMB)^19^.

We set out to address the nucleocytoplasmic shuttling capacity of the HeV P-gene encoded V protein. We show HeV V shuttles between the nucleus and cytoplasm dependent on the importin α1/β1 heterodimer and exportin-1 for nuclear import and nuclear export, respectively; we establish the NES recognized by exportin-1 spans HeV V residues 174–192. Analysis of the conformation of V bound to either nuclear transporter using circular dichroism (CD) and small-angle X-ray scattering (SAXS) reveals the N-terminal 50 residues of V induce α-helicity and compact structure upon binding either partner. Importantly, both ivermectin and LMB reduced HeV infection in mammalian cells. Our findings support the critical role of host nuclear transport apparatus in HeV infection, raising the possibility of using compounds targeting V nuclear transport, such as the FDA-approved agent ivermectin, as anti-HeV agents.

## Results

### HeV V undergoes exportin-1-dependent nuclear export

**Figure 1:**
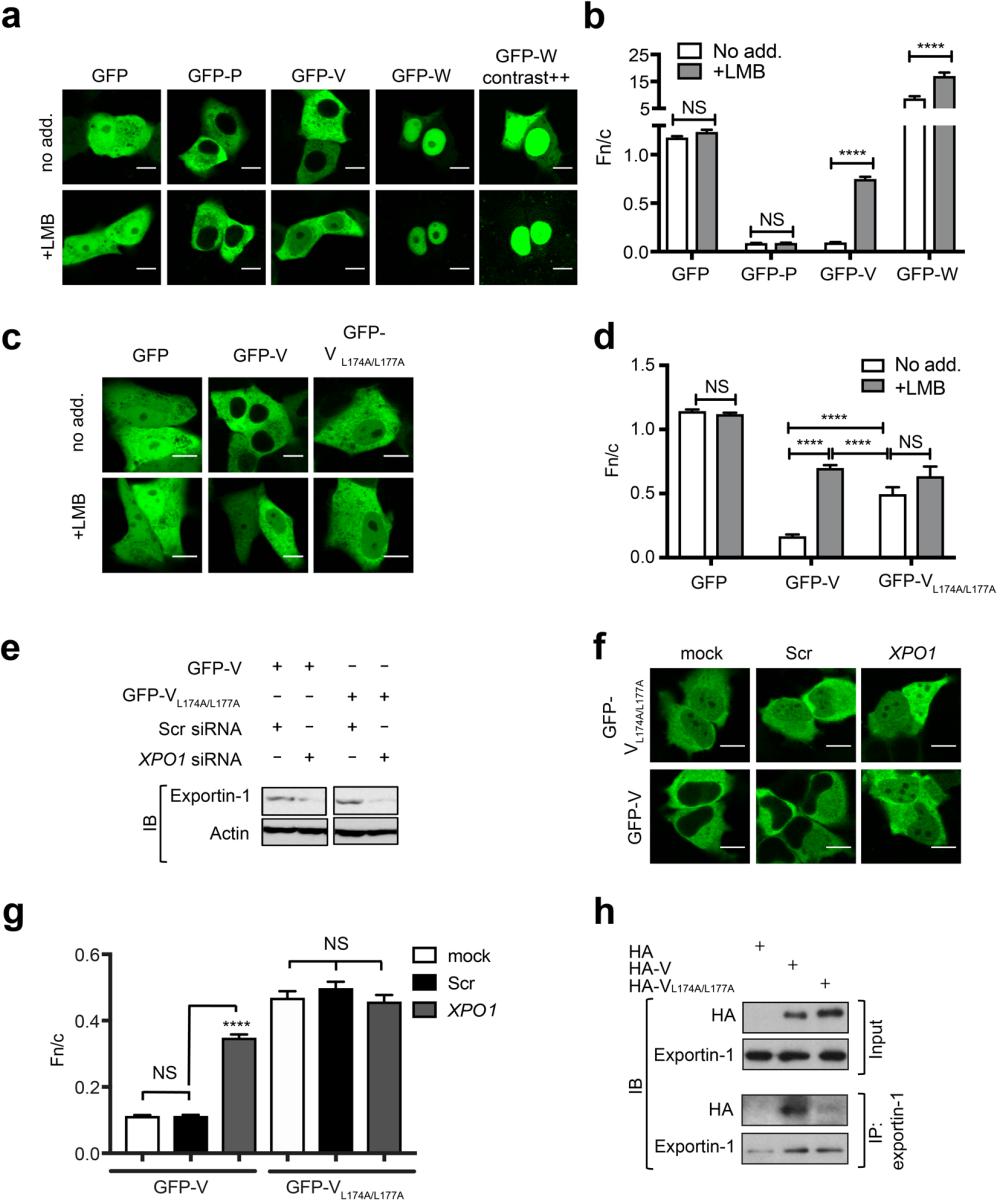
HeV V undergoes NES-dependent, exportin-1 mediated nuclear export. (**a**) Vero cells were transfected to express the indicated GFP-fused HeV proteins (18 h) before treatment with (+) or without (no add.) LMB (3.5 h), then imaged by live-cell CLSM. ‘GFP-W contrast ++’ represents the same cells as for GFP-W, but with enhanced brightness and contrast to allow visualization of cytoplasmic fluorescence. (**b**) Images such as those in (**a**) were analyzed to determine the nuclear/cytoplasmic fluorescence ratio, Fn/c; results represent the mean ± SEM (n ≥ 30 cells) from a single assay representative of three independent assays. ****p < 0.0001; NS, not significant. (**c**) Vero cells were transfected to express the indicated GFP-fused HeV proteins (18 h) before treatment with or without LMB (3.5 h) and live-cell imaging. (**d**) Images such as those in (**c**) were used to determine the Fn/c as per (**b**). (**e–g**) HeLa cells were transfected with scrambled siRNA (Scr) or *XPO1*-targeting siRNA, or mock-transfected (48 h), followed by transfection to express GFP-HeV V or GFP-HeV V_L174A/L177A_ (24 h). (**e**) Exportin-1 and actin expression levels from each sample were determined by western analysis. (**f**) Representative images from live-cell CLSM. (**g**) Images such as those shown in (**f**) were used to calculate the Fn/c as per (**b**); results represent the mean ± SEM (n ≥ 100 cells) from a single assay representative of two independent assays. ****p < 0.0001; NS, not significant. (**h**) HEK293T cells expressing the indicated proteins were subjected to immunoprecipitation using protein G-coupled Dynabeads® with magnetic resin conjugated with anti-exportin-1 antibody, before western blot analysis of cell lysate (input) and immunoprecipitate (IP) using antibodies against HA and exportin-1. Unprocessed original scans of blots can be found in Supplementary Figs 3 and 4. Scale bars represent 10 μM.

A recent genome-wide siRNA screen identified the *KPNA2* (importin α1) and *XPO1* (exportin-1) members of the host nuclear transport machinery as important genes in human HeV infection^20^ (Supplementary Fig. 1). To begin to establish the mechanistic basis for these observations, we set out to investigate the nucleocytoplasmic shuttling capabilities of the HeV P-gene encoded products, in part, in light of the report of an exportin-1 recognized nuclear export signal (NES) in NiV V^21^ that is conserved in HeV V. Vero cells were initially transfected to express GFP-fused HeV P, V or W proteins, before treatment without or with LMB, imaged by live-cell confocal laser scanning microscopy (CLSM) and the nuclear/cytoplasmic fluorescence ratio (Fn/c) was calculated (Fig. 1a,b). In untreated cells, HeV P and V were excluded from the nucleus, whereas W showed strong nuclear localization, but was excluded from structures consistent with nucleoli (Fig. 1a,b). Following LMB treatment HeV V showed a marked increase in nuclear localization (p < 0.0001), but remained excluded from nucleoli (Fig. 1a,b), providing the first empirical evidence that HeV V undergoes exportin-1-dependent nuclear export. As per V, the nuclear localization of HeV W also increased following LMB treatment (p < 0.0001) (Fig. 1a,b), but in contrast the localization of HeV P remained unchanged following LMB treatment (Fig. 1a,b). Thus, the nucleocytoplasmic localization of HeV V and W, but not P, is dependent on an exportin-1-dependent nuclear export sequence (NES).

### Nuclear export of HeV V is dependent on residues L174 and L177

Based on homology with the NES of NiV V^21^ the full HeV V NES likely spans residues 174–192. Based on the consensus for exportin-1-recognized NESs and previous studies of NiV V^21^, we decided to substitute the leucines at positions 174 and 177 with alanines to confirm the key residues of the HeV V NES. In untreated cells the HeV V double leucine mutant (V_L174A/L177A_) showed a significant increase (p < 0.0001) in nuclear localization compared to wild-type V (Fig. 1c,d), suggesting loss of exportin-1-mediated nuclear export of V. Consistent with this, HeV V_L174A/L177A_ nuclear localization was not strongly affected by LMB treatment (Fig. 1c,d). Together, these data indicate that exportin-1-mediated nuclear export of HeV V is dependent on residues L174 and L177, and that the NES from NiV V is conserved in HeV V.

### siRNA knockdown of exportin-1 increases nuclear accumulation of HeV V

To demonstrate the role of exportin-1 in the nuclear export of HeV V, we used siRNA to knockdown exportin-1 expression. HeLa cells were mock-transfected, or transfected with siRNA targeting *XPO1* (exportin-1) or scrambled (Scr) siRNA, prior to transfection to express wild-type HeV V or V_L174A/L177A_. Exportin-1 expression was reduced by approximately 70% in cells transfected with *XPO1* siRNA compared with Scr siRNA (Fig. 1e, Supplementary Figs 2 and 3). A significant (p < 0.0001) increase in the extent of nuclear accumulation of wild-type HeV V was observed in cells transfected with *XPO1* siRNA, compared to mock-treated cells and those transfected with Scr siRNA (Fig. 1f,g). In contrast, there was no significant effect of *XPO1* siRNA on the localization of HeV V_L174A/L177A_ (Fig. 1f,g), consistent with the idea that L174 and L177 are essential for exportin-1-mediated nuclear export of HeV V.

Consistent with the above results, coimmunoprecipitation revealed endogenous exportin-1 could readily interact with HA-fused wild-type HeV V, but the interaction with HA-tagged V_L174A/L177A_ appeared reduced in comparison (Fig. 1h, Supplementary Fig. 4). Taken together, our data indicate that HeV V undergoes nuclear export through a conventional leucine-rich NES-, exportin-1-dependent mechanism.

### HeV V can undergo importin α1/β1-dependent nuclear import

**Figure 2:**
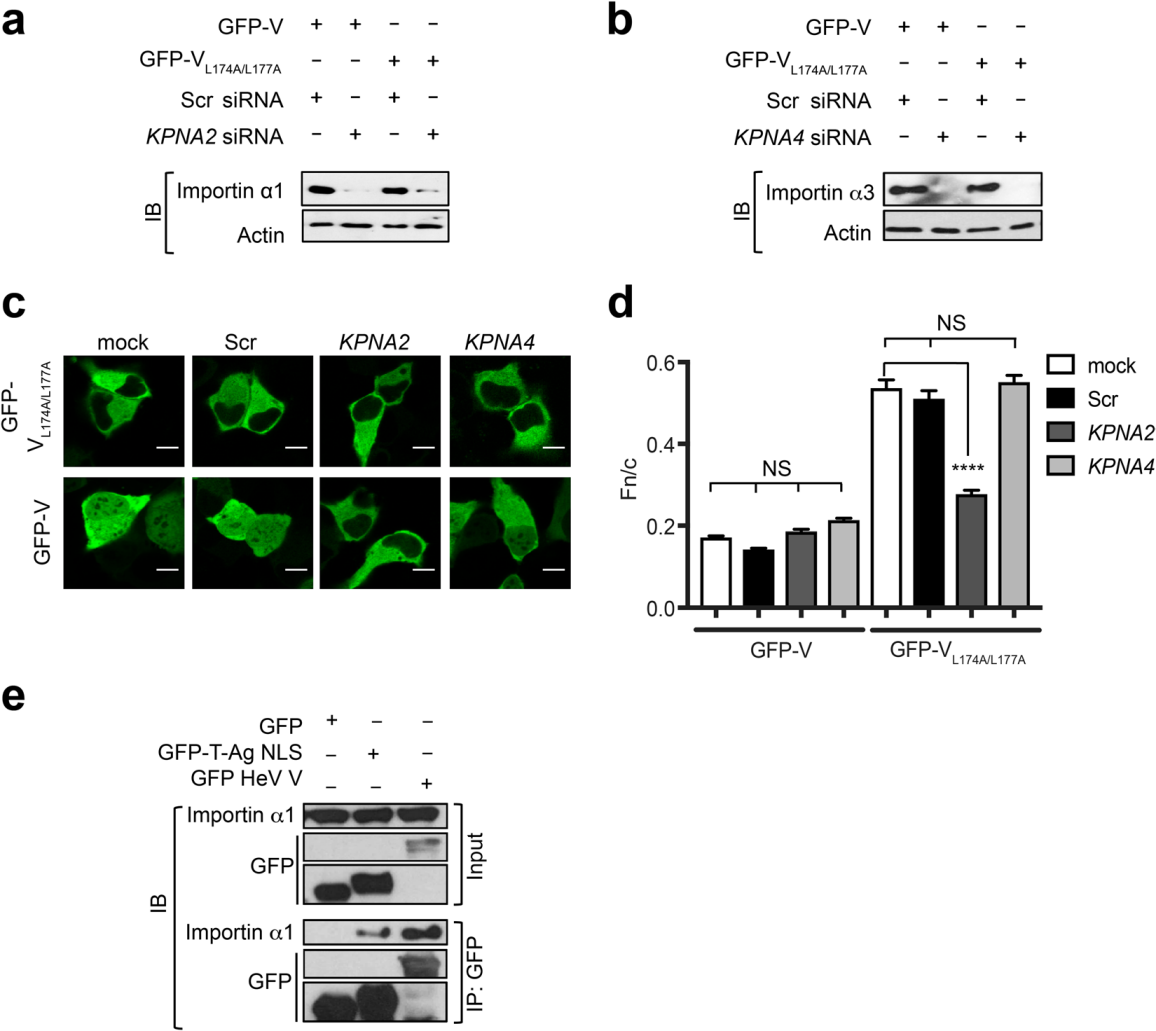
HeV V undergoes importin α1-dependent nuclear import. HeLa cells were transfected with siRNAs targeting *KPNA2* (encoding importin α1), *KPNA4* (encoding importin α3) siRNA, or control (scrambled - Scr) siRNA or mock-transfected (48 h), followed by transfection to express GFP-HeV V or GFP-HeV V_L174A/L177A_ (24 h), then imaged by live-cell CLSM. (**a**) Importin α1, (**b**) importin α3 and actin expression levels were determined by western analysis. (**c**) Representative images from live-cell CLSM. Scale bars represent 10 μM. (**d**) Images such as those shown in (**c**) were used to calculate the Fn/c; results represent the mean ± SEM (n ≥ 100 cells) from a single assay representative of two independent assays. ****p < 0.0001; NS, not significant. (**e**) HEK293T cells expressing the indicated GFP-fusion proteins were subjected to immunoprecipitation using GFP-Trap® before western analysis of cell lysate (input) and immunoprecipitate (IP) using antibodies against GFP and importin α1. Unprocessed original scans of blots can be found in Supplementary Figs 5 and 6.

That HeV V relocalizes to the nucleus following LMB treatment (Fig. 1a) implies an active importin-dependent nuclear import mechanism. Since one of the importin α nuclear import proteins (*KPNA2*) is critical for HeV infection, as implicated by siRNA screening (see Supplementary Fig. 1)^20^, we tested whether HeV V may have an importin α1/β1-dependent nuclear import mechanism. Accordingly, GFP-fused HeV V wild-type or V_L174A/L177A_ were expressed in HeLa cells either mock-transfected or transfected with siRNAs specific for *KPNA2* (importin α1) or *KPNA4* (importin α3), or a scrambled (Scr) control. Western blot analysis confirmed specific knockdown of importin α1 and α3 (>90%), respectively, in cells transfected with *KPNA2* and *KPNA4* siRNAs (Fig. 2a,b, Supplementary Figs 2 and 5), but the extent of nuclear accumulation of wild-type HeV V was not reduced in cells transfected with any of the siRNAs, compared with mock-transfected cells (Fig. 2c,d). This was expected due to the strong cytoplasmic localization of V at steady state, driven principally by the NES. To examine nuclear import of HeV V directly, the double leucine mutant (V_L174A/L177A_) was used, in which the NES is largely inactivated. While there was no significant effect on the nuclear accumulation of HeV V_L174A/L177A_ upon importin α3 knockdown, importin α1 knockdown caused a significant (p < 0.0001) decrease in nuclear accumulation (Fig. 2c,d).

Consistent with this result, GFP-fused V coimmunoprecipitated endogenous importin α1 to levels comparable to those of a GFP-fusion protein containing the prototypic importin α/β1-recognized NLS from simian virus (SV40) large tumour antigen GFP-T-Ag NLS (Fig. 2e, Supplementary Fig. 6). Taken together, results suggest HeV V shuttles dynamically between the host cell nucleus and cytosol, accessing the nucleus by subverting importin α1 of the host nuclear transport system.

### Exportin-1/Ran-GTP and importin α2/β1 directly interact with HeV V

**Figure 3:**
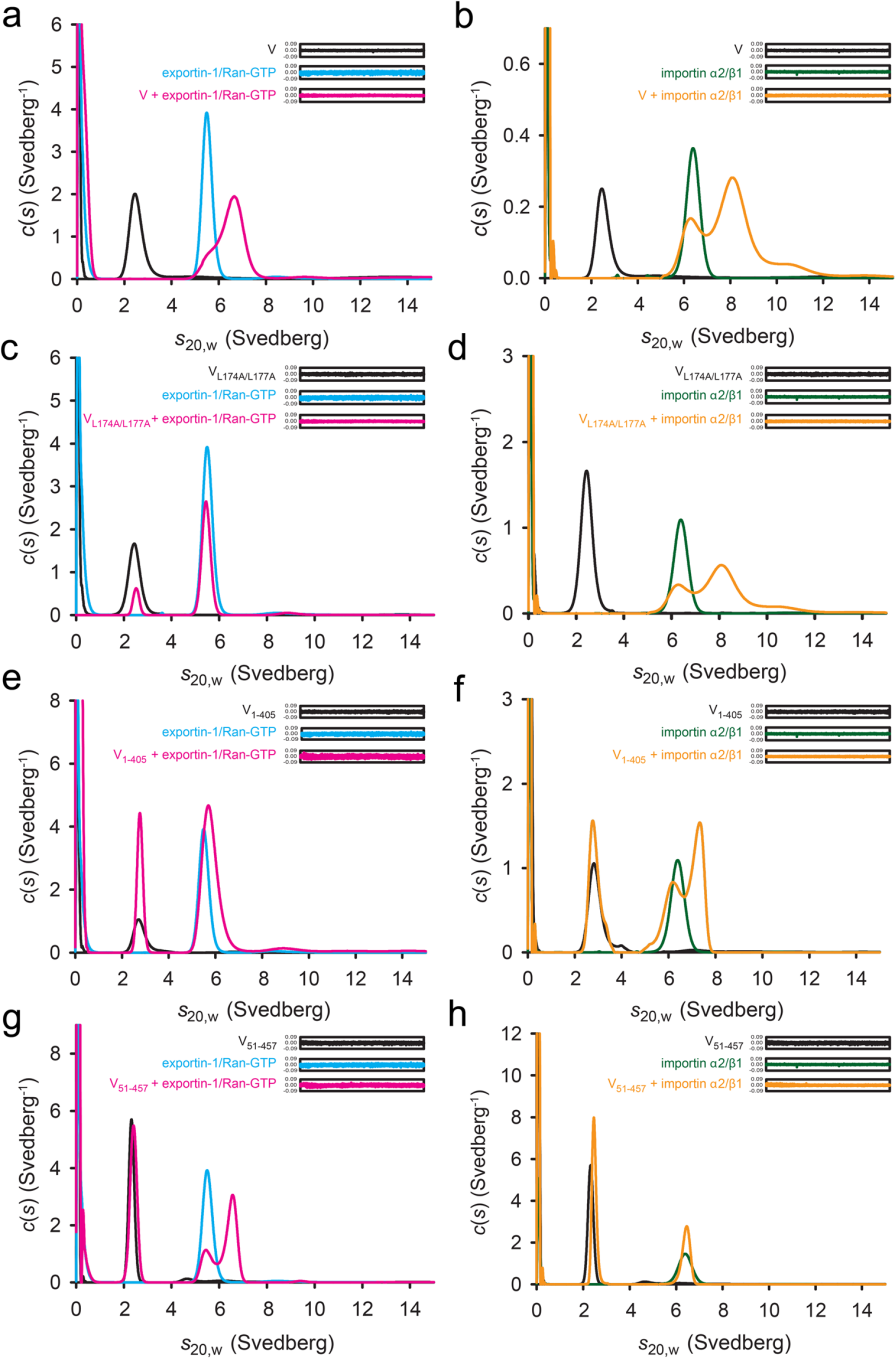
Analytical ultracentrifugation reveals direct binding of exportin-1 and importin α2/β1 to HeV V. Sedimentation velocity analytical ultracentrifugation experiments were performed on purified recombinant exportin-1/Ran-GTP, importin α2/β1, wild-type HeV V and variants thereof, both alone and in various combinations. The continuous sedimentation coefficient distribution [(*c*)*s*] was plotted as a function of *s*_20,w_ (black) for HeV V (**a,b**) wild-type, (**c,d**) V_L174A/L177A_, (**e,f**) V_1-406_, and (**g,h**) V_51-457_. Exportin-1/Ran-GTP (blue) and importin α2/β1 (green) are shown. Equimolar V:exportin-1/Ran-GTP mixtures are shown in magenta and V:importin α2/β1 mixtures are shown in orange. Residual plots shown in insets.

To confirm whether the interaction of HeV V with the nuclear transporters is direct, we analyzed binding of purified, tagless recombinant proteins by analytical ultracentrifugation sedimentation velocity experiments (Fig. 3a,b); in the case of human importin α1/β1, the mouse homologue α2/β1 was used in which the α- and β- chains have 94.5% and 99.2% identity, respectively. HeV V sedimented as a single species with a sedimentation coefficient (*s*_20,w_) of 2.5 S, whereas the larger importin α2/β1 and exportin-1/Ran-GTP complexes showed sedimentation coefficients (*s*_20,w_) of 6.4 S and 5.1 S, respectively (Supplementary Table 1). The formation of a larger sedimenting species when HeV V was incubated with equimolar concentrations of importin α2/β1 (8.1 S) or exportin-1/Ran-GTP (6.7 S) confirmed HeV V bound either importin α2/β1 and exportin-1/Ran-GTP directly (Fig. 3a,b and Supplementary Table 1).

We assessed the ability of importin α2/β1 and exportin-1/Ran-GTP to bind the V nuclear export mutant (V_L174A/L177A_), V bearing an N-terminal deletion (V_51–457_) to remove a region of predicted secondary structure^6,7^, and the predominantly disordered N-terminal region (V_1-405_). As expected, the absence of a larger sedimenting species when V_L174A/L177A_ was incubated with exportin-1/Ran-GTP (Fig. 3c) confirmed that L174 and L177 within the HeV V NES are critical to exportin-1/Ran-GTP binding. In contrast, binding was observed when both V_1-405_ (Fig. 3e) or V_51-457_ (Fig. 3g) were mixed with exportin-1/Ran-GTP, indicating that neither the C-terminal region (residues 406–457) nor N-terminal 50 residues of V are critical for exportin-1/Ran-GTP binding. Rather than the presence of a clear third peak as for the other complexes, a shift in sedimentation coefficient was observed in Fig. 3e, suggestive of a weaker interaction. Thus while the C-terminal region is not essential for V:exportin-Ran-GTP binding, it contributes markedly to binding.

In contrast to exportin-1/Ran-GTP, importin α2/β1 incubation with HeV V_L174A/L177A_ yielded a larger sedimenting species consistent with the formation of a V:importin α2/β1 complex, confirming as expected, the NES is not required for importin binding (Fig. 3d). Similarly, we observed binding when V_1–405_ was incubated with importin α2/β1 (Fig. 3f), but not upon incubation of V_51-457_ with importin α2/β1 (Fig. 3h). This suggests that the first 50 residues of V are required for interaction with importin α2/β1. Taken together, our data suggest that the residues required for direct binding of HeV V to exportin-1/Ran-GTP (L174/L177) and importin α2/β1 all reside within the N-terminal region of HeV V.

### HeV V undergoes induced folding upon binding exportin-1/Ran-GTP and importin α2/β1

A previous study showed the shared N-terminal region of HeV P, V and W (residues 1-405) is largely disordered^7^. Using CD we observed full-length HeV V displayed a single minimum at around 200 nm, which is indicative of a largely unstructured protein. Also apparent was broad but weak negative ellipticity between 215 and 235 nm, indicative of a small proportion of residual secondary structure that is absent in the CD spectra of V_1-405_. Based on this data we concur HeV V_1-405_ is predominantly intrinsically disordered (Supplementary Fig. 7a).

Many IDPs and IDRs undergo disorder-to-order transitions upon engagement with physiological partners^22–24^. The same has been presumed for HeV V based on the α-helical structure HeV V gains in the presence of the solvent 2,2,2-trifluoroethanol (TFE)^7^, but has not been tested using physiological binding partners. Thus, we performed far-UV CD spectroscopy binding studies between V and exportin-1/Ran-GTP or importin α2/β1 to visualize possible HeV V structural transitions that occur upon binding. We compared the observed spectra (obs) with the calculated theoretical average curves (calc) expected for the proteins in complex (ref.^25^, see Methods). Deviations between the calculated and observed spectra for the complexes suggest structural transitions.

**Figure 4:**
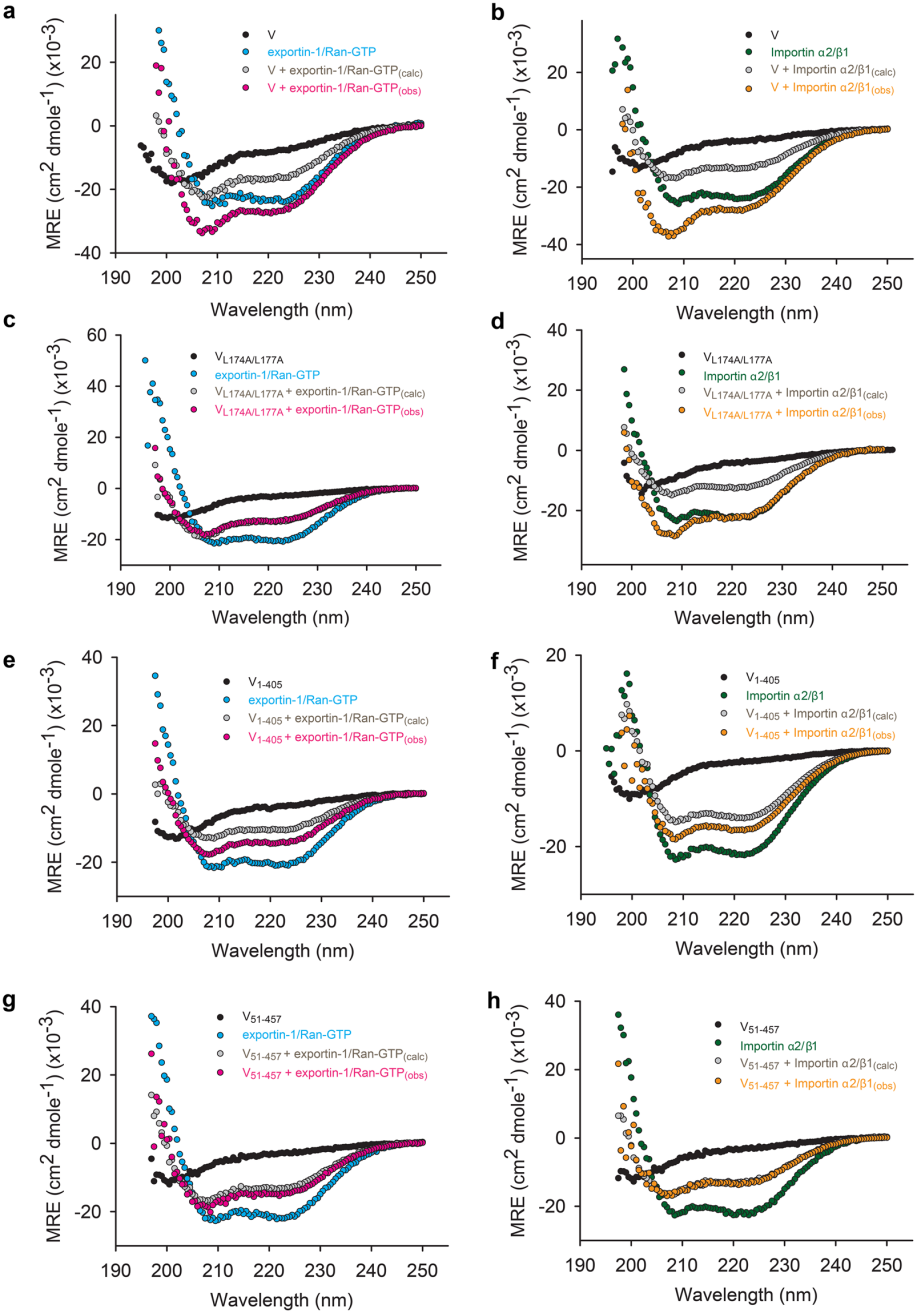
Exportin-1/Ran-GTP and importin α2/β1 induce folding of wild-type HeV V and V variants. CD spectra of (**a,b**) V, (**c,d**) V_L174A/L177A_, (**e,f**) V_1-405_, and (**g,h**) V_51-457_ are shown in black, with the spectra of (**a,c,e,g**) exportin-1/Ran-GTP and (**b,d,f,h**) importin α2/β1 in blue and green, respectively. Spectra of equimolar mixtures (obs) of V:importin α2/β1 (orange) and V:exportin-1/Ran-GTP (magenta) are shown. Theoretical average curves (calc) calculated by assuming that no structural variations occur (see Methods) are shown in gray.

The CD spectra for exportin-1/Ran-GTP, importin α2/β1 and a bovine serum albumin (BSA) control displayed a double minimum at 208 nm and 222 nm (Fig. 4a,b and Supplementary Fig. 7b), in agreement with their respective α-helical crystal structures^26–28^. The CD spectrum of HeV V was once again characteristic of a largely unstructured protein (Fig. 4a,b).

Exportin-1/Ran-GTP, importin α2/β1 or BSA were subsequently mixed with HeV V in equimolar proportions (Fig. 4a,b and Supplementary Fig. 7b). When mixed with exportin-1/Ran-GTP or importin α2/β1, HeV V displayed a random coil-to-α-helix transition, as indicated by the more pronounced minima at 208 and 222 nm of the experimentally observed spectrum (Fig. 4a magenta, Fig. 4b orange) compared with the corresponding calculated theoretical average curves (Fig. 4a,b, gray). Moreover, the α-helical content of the mixtures (68% for V:exportin-1/Ran-GTP and 71% for V:importin α2/β1) are not only higher than that of the theoretical curves, but are also higher than the α-helical content of exportin-1/Ran-GTP or importin α2/β1 alone (58% and 61% respectively). In contrast, the CD spectrum of HeV V mixed with BSA was superimposable on the corresponding theoretical average curve (Supplementary Fig. 7b yellow vs gray), indicating no structural changes occurred, as consistent with a lack of interaction between the two proteins. Taken together, our results are consistent with HeV V undergoing an interaction-dependent gain of structure indicative of a disorder-to-order transition upon binding to exportin-1/Ran-GTP or importin α2/β1.

### HeV V residues 1–50 are required for V to gain order upon exportin-1/Ran-GTP binding

To gain insights into structural transitions that occur upon HeV V binding to exportin-1/Ran-GTP we repeated the CD spectroscopy studies described above, with our set of HeV variant proteins (Fig. 4c,e,g). Of the three V variants only V_1-405_ underwent a random coil-to-α-helix transition upon binding exportin-1/Ran-GTP (Fig. 4e), as indicated by the more pronounced minima at 208 and 222 nm of the experimentally observed spectrum (magenta) compared with the corresponding calculated theoretical average curve (gray), and in accordance with binding having occurred in the first place (Fig. 3e). This gain in order was less prominent than for full-length V (Fig. 4e vs Fig. 4a), implying that V residues 406–457 may also contribute to the structural transition upon binding with exportin-1/Ran-GTP, but are not necessary for binding (Fig. 3e). In contrast, V structural gain was negligible upon mixing V_L174/L177A_ or V_51-457_ with equimolar exportin-1/Ran-GTP (Fig. 4c,g magenta vs gray). This was expected for V_L174/L177A_ since it does not bind to exportin-1/Ran-GTP (Fig. 3c), but not for V_51-457_, since the first 50 residues are dispensable for exportin-1/Ran-GTP binding (Fig. 3g). These studies suggest that HeV V residues 1–50, which are the only residues with predicted secondary structure in the shared P/V/W-N-terminal region, are critical to the disorder-to-order transition of V upon binding to exportin-1/Ran-GTP.

### HeV V residues 1–50 are essential for importin α2/β1 binding

Next, we performed far-UV CD spectroscopy binding studies for the various V variants with importin α2/β1. As expected, the V_L174A/L177A_ variant that contained a NES substitution that is irrelevant to importin α2/β1 binding, underwent structural changes in the presence of importin α2/β1 (Fig. 4d gray vs orange) comparable to wild-type V (Fig. 4b gray vs orange). V_1-405_ also underwent a small structural change following importin α2/β1 binding, although the change was not as pronounced as for full-length V (comparison of gray vs orange in Fig. 4f and b), implying that the V C-terminal 52 residues contribute to the structural transitions upon binding to importin α2/β1, but are not essential for binding (Fig. 3f). As expected, V_51-457_ did not undergo structural change when mixed with importin α2/β1 (Fig. 4h, gray vs orange), consistent with an inability to bind importin α2/β1 (see Fig. 3h). This result confirms the first 50 residues of HeV V are required for binding to importin α2/β1 and most of the resultant interaction-dependent gain of order.

### HeV V becomes more compact upon binding exportin-1/Ran-GTP and importin α2/β1

**Figure 5:**
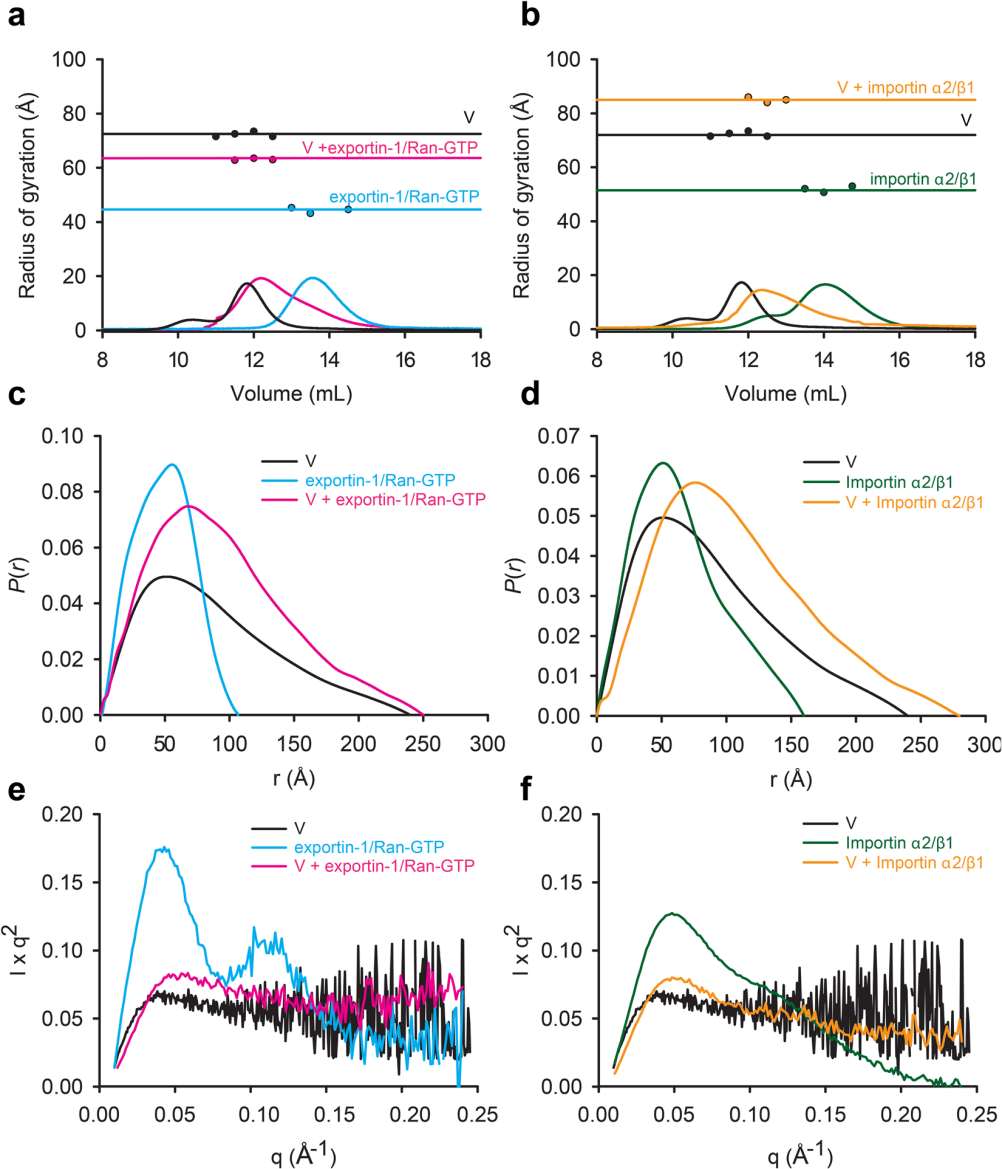
HeV V adopts a more compact conformation upon exportin-1/RanGTP or importin α2/β1 binding as determined by SAXS analysis. (**a**) Size exclusion chromatography profiles for the V:exportin-1/Ran-GTP SAXS experiment. *R*_g_ values of HeV V, exportin-1/Ran-GTP and the V:exportin-1/Ran-GTP complex were calculated across each peak, with average values of 72.0 ± 0.5 Å, 44.0 ± 0.6 Å and 64 ± 0.2 Å, respectively, shown by horizontal lines. (**b**) Size exclusion chromatography profiles for the V:importin α2/β1 SAXS experiment. Radius of gyration (*R*_g_) values of HeV V, importin α2/β1 and the V:importin α2/β1 complex were calculated across each peak, with average values of 72.0 ± 0.5 Å, 52.0 ± 0.7 Å and 85.0 ± 0.6 Å, respectively, shown by horizontal lines. (**c**) *P*(*r*) plots for HeV V, exportin-1/Ran-GTP and the V:exportin-1/Ran-GTP complex. (**d**) *P*(*r*) plots for HeV V, importin α2/β1 and the V:importin α2/β1 complex. Kratky plots show that unlike (**e**) exportin-1/Ran-GTP and (**f**) importin α2/β1, V shows no globular fold. Some lack of structure is retained in the V:exportin-1/Ran-GTP and V:importin α2/β1 complexes.

To gain further insight into the size and shape of HeV V, as well as the V:exportin-1/Ran-GTP and V:importin α2/β1 complexes in solution, we performed size exclusion chromatography in-line with small-angle X-ray scattering (SEC*-*SAXS) on purified, tagless proteins (Supplementary Fig. 8). Guinier plots were calculated for HeV V, exportin-1/Ran-GTP and importin α2/β1, revealing the radius of gyration (*R*_g_) for the protein/protein complexes to be 72 Å, 44 Å and 52 Å, respectively (Fig. 5a,b). Despite being the smallest of the three proteins (Supplementary Table 1), HeV V has the largest *R*_g_, indicating its structure has the least compact conformation. Guinier analysis of the V:nuclear transport complexes revealed V adopts a much more compact conformation upon complex formation, with *R*_g_ values of 64 Å and 85 Å for the V:exportin-1/Ran-GTP and V:importin α2/β1 complexes, respectively.

The distance distribution function, or *P*(*r*), was used to calculate the largest particle dimension (*D*_max_) of all components in their un/bound state (Fig. 5c,d). The *D*_max_ of the V:exportin-1/Ran-GTP complex is 250 Å, whereas free V and exportin-1/Ran-GTP had maxima of 240 Å and 120 Å, respectively (Fig. 5c). The 130 Å difference between the *D*_max_ of exportin-1/Ran-GTP and the V:exportin-1/Ran-GTP complex, is much smaller than that of V alone (240 Å), and in addition to the reduced *R*_*g*_ value of V:exportin-1/Ran-GTP indicates that binding of exportin-1/Ran-GTP dramatically increases the folded nature of most of the length of V (Fig. 5a). Similarly, based on the *D*_max_ values of V (240 Å) and importin α2/β1 (155 Å) alone, the observed *D*_max_ of 270 Å was much lower than the anticipated *D*_max_ of the V:importin α2/β1 complex of approximately 395 Å, suggesting that V also adopted a more compact conformation upon binding importin α2/β1 (Fig. 5d). Given the high *D*_max_ of HeV V, and to rule out aggregation, we performed sedimentation velocity analytical ultracentrifugation analysis of V at a similar concentration (0.45 mg/ml) to that eluting in the SEC-SAXS experiments (Supplementary Fig. 9). We confirmed that HeV V exists predominantly as a monomer in solution, with a sedimentation coefficient (*s*_20,w_) of 2.5 S and molecular mass of 50.3 kDa (Supplementary Fig. 9; Supplementary Table 1).

Kratky plots were used to further assess the conformational state of V bound to either exportin-1/Ran-GTP or importin α2/β1 in solution (Fig. 5e,f). In the unbound state both exportin-1/Ran-GTP (Fig. 5e, blue) and importin α2/β1 (Fig. 5f, green) displayed a bell-shaped curve with a well-defined maximum followed by a drift to baseline, typical of globular folded proteins and consistent with the solved X-ray structures of these proteins^27–29^. In contrast, the Kratky plot of V (Fig. 5e,f, black) lacked a well-defined peak and plateaued at high *s* values, as is typical of an unfolded protein. Strikingly, the curves for the V:exportin-1/Ran-GTP and V:importin α2/β1 complexes displayed characteristics of both folded and unfolded proteins, which suggested both complexes are partially folded and retain some disorder (Fig. 5e,f, magenta and orange, respectively).

Collectively these data suggest that upon HeV V binding to exportin-1/Ran-GTP or importin α2/β1 the maximum distance occupied by V is significantly reduced, indicating binding-induced structural compaction. This is likely due to a gain in V α-helicity as suggested by our CD binding studies (Fig. 4). Notably, when bound to either transport protein, V retained residual disorder.

### Small molecule inhibitors that block high affinity recognition of HeV V by exportin-1/Ran-GTP or importin α2/β1 reduce HeV infection

**Figure 6:**
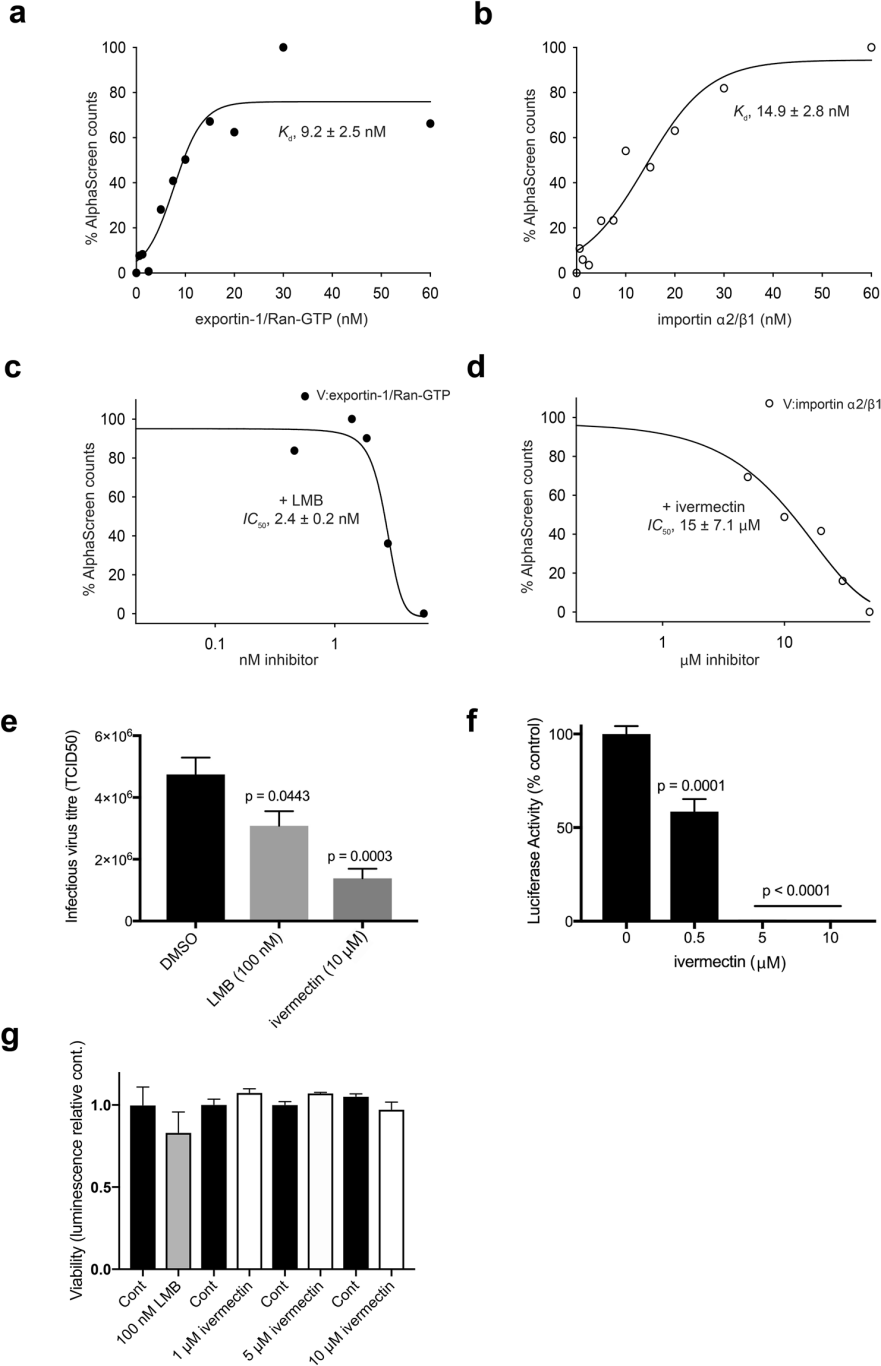
Inhibitors that block high affinity recognition of HeV V by importin α2/β1 or exportin-1/Ran-GTP can reduce HeV production. (**a**) HeV V binds exportin-1/Ran-GTP and (**b**) importin α2/β1 with nM binding affinity. 30 nM biotinylated HeV V was incubated with increasing concentrations of His-fused exportin-1/Ran-GTP or importin α2/β1. Results are expressed as a percentage of AlphaScreen counts relative to maximal binding, with apparent dissociation constants (*K*_*d*_ ± SEM, n = 3) indicated. LMB and ivermectin inhibit HeV V binding to (**c**) exportin-1/Ran-GTP and (**d**) importin α2/β1, respectively. HeV V (30 nM) was added to importin α2/β1 (30 nM) or exportin-1/Ran-GTP (20 nM) complexes followed by increasing concentrations of inhibitors. Addition of 5.5 nM LMB or 50 µM ivermectin did not inhibit HeV V binding to importin α2/β1 or exportin-1/Ran-GTP, respectively. Results are expressed as a percentage of AlphaScreen counts relative to no inhibitor, with the IC_50_ values (mean ± SD, n = 2) indicated. (**e**,**f**) Inhibition of HeV infection by LMB or ivermectin. (**e**) Vero cells were either pretreated for 3 h with the indicated concentrations of ivermectin prior to medium change and infection (ivermectin was re-added after infection), or treated with 100 nM LMB at the time of infection with a wild-type HeV. Production of infectious virus was measured by TCID_50_ at 24 h post-infection (p.i.) as previously^66^; results represent the mean ± SEM (n = 8) for a single typical experiment from a series of 3 similar experiments. (**f**) Vero cells treated/pretreated with ivermectin as per (**e**) were infected with HeV encoding a firefly luciferase reporter. Cells were lysed 24 h p.i. and luciferase activity measured as described in Methods; results represent the mean ± SEM (n ≥ 6) for a single typical experiment from a series of 2 similar experiments, expressed as % luciferase activity relative to the DMSO-treated control (**e**). (**g**) Vero cells treated with inhibitors as per (**e**,**f**) were analyzed for cytotoxicity using CellTiter-Glo® (Promega) as described in Methods. Results represent the mean ± SEM (n ≥ 3).

Given that HeV V can interact with exportin-1/Ran-GTP and importin α2/β1 directly, we tested the ability of known inhibitors of exportin-1 and importin α1/α2, LMB and ivermectin, respectively, to inhibit these interactions using an established AlphaScreen binding assay^30^. Results indicated direct high affinity (low nM) binding of either transport complex to HeV V (Fig. 6a,b), consistent with the findings in Figs 4 and 5. Importantly, LMB inhibited binding to HeV V of exportin-1/Ran-GTP (IC_50_ of approximately 2.4 nM) (Fig. 6c,d), while ivermectin inhibited binding of importin α2/β1 (IC_50_ of approximately 15 μM) (Fig. 6c,d), consistent with results from previous studies with other viral proteins^18,31^.

Since *KPNA2* and *XPO1* are pivotal to human HeV infection^20^ we tested whether LMB or ivermectin could inhibit HeV infection in mammalian cells. Significantly, both LMB and ivermectin could reduce HeV production (Fig. 6e); even when used at a non-optimized single dose of 10 μM, ivermectin reduced production of the field strain by almost 5-fold. Lower concentrations also inhibited HeV production using a luciferase HeV-reporter system (Fig. 6f). Importantly, ivermectin showed no toxicity at a concentration of 10 μM, consistent with other studies (CC50 of 150 μM)^18,30^. The rigors of performing infectious assays with HeV under high security conditions prevented formal IC50 analysis for ivermectin and HeV infection, but the results in Fig. 6f enable extrapolation of a value of c. 2 μM, meaning that the selectivity index of ivermectin is of the order of >70. We also performed analysis for the importin α1 targeting agent Gossypol (GSP), which is structurally very different from ivermectin but identified in the same high throughput screen that originally identified ivermectin^30^, results showing clear inhibition of HeV infection at 10 μM without cytotoxicity (Supplementary Fig. 10a,b). Like ivermectin, GSP was found to clearly inhibit V:importin α2/β1 complex formation in sedimentation velocity experiments (Supplementary Fig. 10c,d). Formation of the V:importin α2/β1 complex (see Fig. 3b) was reduced markedly by ivermectin (Supplementary Fig. 10c), concomitant with higher proportions of unbound importin α2/β1 (6.4 S) and HeV V (2.5 S) species. GSP similarly effected a reduction in V:importin α2/β1 complex formation, and the generation of larger species with higher s-values, suggesting the formation of larger complexes unable to undergo nuclear import (Supplementary Fig. 10d). That two chemically distinct agents able to inhibit HeV V interaction with importin α1/β1 also inhibit HeV infection clearly supports the idea that HeV nuclear import is critical for HeV infection, and that nuclear transport inhibitors such as the FDA-approved ivermectin could productively be used to treat HeV infection.

## Discussion

HeV infection is deadly to humans, with sporadic outbreaks increasing in frequency, and the geographical distribution of the disease likely to widen in the future, highlighting the need for agents to treat HeV infection. Paramount to this is detailed understanding of HeV-host interactions that may enable development of novel therapeutic strategies.

Although their genome replication occurs in the cytoplasm of infected cells, paramyxoviruses have evolved intricate mechanisms to exploit host cell machinery to enable specific viral proteins to access the nucleus. HeV appears to be no exception, and here we show for the first time that HeV V undergoes active nuclear import and nuclear export that is dependent on importin α1/β1 and exportin-1, respectively. It would obviously be important to confirm our inhibitor studies by immunostaining for V in HeV infected cells, but this will require the future development of anti-V antibodies suitable for immunostaining^32^.

Despite the fact that P, V and W share the exportin-1 recognized NES shown to be active for V here, there are inherent differences in the steady-state localization of the P-gene encoded products, whereby V is predominantly cytoplasmic and W is predominantly nuclear, implying the unique C-terminal regions of these proteins contribute strongly to nucleocytoplasmic trafficking (see^16,33^).

The shared N-terminal region of HeV P/V/W is predominantly an IDR. As IDPs/IDRs are dynamic they impede high-resolution structure determination and therefore alternate tools are required to describe their shape and mechanism of molecular recognition. Results presented here show that the largely disordered HeV V gains α-helical structure, and becomes more compact, upon binding its novel host cell binding partner complexes, the mouse homologue of importin α1/β1 (importin α2/β1) and exportin-1/Ran-GTP. It is of interest to determine whether the V disorder-to-order transition is specific to binding members of the importin superfamily, or may apply with other V interaction partners. In this context, interaction between HeV V:MDA5 is mediated by the conserved, structured V-CTD^9,10^, making this interaction clearly dissimilar to that of HeV V-importin/exportin-1 which is mediated predominantly by the largely disordered V-NTD. Importantly, the HeV:STAT1 interaction is dependent on the V-NTD^11^, but our *in vitro* analysis indicates lack of interaction of HeV V with recombinant STAT1, implying that interaction requires other factors and/or posttranslational modification of STAT1.

**Figure 7:**
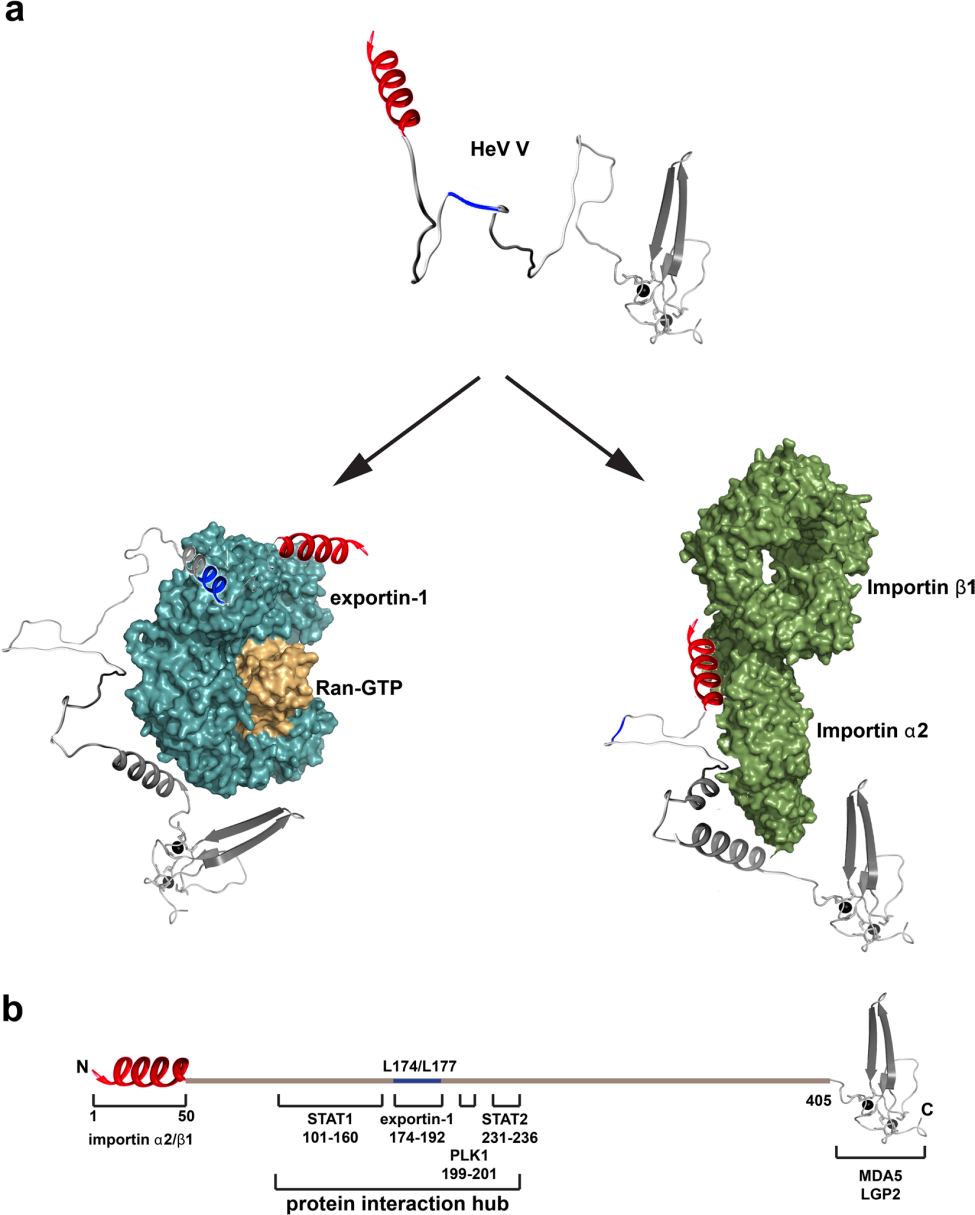
Model of HeV V disorder-to-order transition upon binding exportin-1/Ran-GTP or importin α2/β1. (**a**) Structural model of HeV V with the N-terminal 50 residues predicted to form α-helical structure shown in red, and the NES, which is typically a helix, shown in blue. Models are also shown of V alone and V in complex with exportin-1/Ran-GTP (constructed from PDB ID 3NC1) and importin α2/β1 (constructed from PDB IDs 1EJL and 4XRK). The V model is based on our CD and SAXS data. The V:nuclear transport complex models are based on our data indicating that residues L174/L177 within the V NES are critical for binding the NES groove of exportin-1, whereas residues 1–50 are critical for importin α2/β1 binding. Following binding residues 1–50 enable V to gain α-helicity, leading to V compaction, although the complexes retain residual disorder. (**b**) Schematic of the architecture of HeV V and regions involved in molecular recognition based on our study and mapping studies conducted with HeV V, or the closely related NiV V.

We pinpoint two residues within the V NES, leucine 174 and leucine 177, that are essential for effective interaction with exportin-1/Ran-GTP, while residues 1–50, although not required for binding *per se*, are critical in triggering a gain in V α-helicity that results in V compaction. Likewise, residues 1–50 are also coupled to the folding of V upon importin α2/β1 binding, but in this instance, these residues also appear to be essential. V residues 1–50 thus trigger folding of V upon binding to either importin α2/β1 or exportin-1/Ran-GTP, lending support the region harbours residual structure, as this is considered important for the folding upon binding of IDPs^34^ (Fig. 7a). This 50 amino acid stretch encompasses two short sequence motifs, soyuz1 (residues 2–17) and soyuz2 (residues 27–46), that are conserved in all, or some, *Paramyxovirinae*, respectively^35^. Consequently soyuz1 is predicted to be vital to viral replication, perhaps by binding a conserved viral or host partner, whereas soyuz2, which is conserved in some *Paramyxovirinae*, including HeV V, has been suggested to bind a cellular partner necessary for STAT1 inhibition^35^. The present study raises the intriguing possibility that importin α1/β1 could be this host cellular factor, as the region encompassing both soyuz1 and soyuz2 in HeV V is critical to both binding the mouse homologue of importin α1/β1 (importin α2/β1) and interaction-dependent V folding. Furthermore, nuclear import can permit viral proteins, such as the IFN agonist V, access to nuclear host factors such as phosphorylated STAT1.

Although HeV V gains order upon binding both importin α2/β1 and exportin-1/Ran-GTP, in both cases it retains residual disorder and forms residually disordered, or fuzzy, complexes^36^. V malleability may fine-tune binding to discriminate between multiple interaction partners^37^. It may also assist the transport of V through the NPC in similar fashion to what appears to be the case for importin β1 itself^38–40^, as well as allowing for faster dissociation to bind other host proteins to subvert antiviral functions. Intriguingly *Henipavirus* proteins are renowned for forming fuzzy complexes following partner recognition^41–43^, a phenomenon that extends to other *Paramyxoviridae*^44,45^ and also to other virus families^46–48^, although the physiological importance is unclear.

Through our results here for importin α1/β1 and exportin-1, the number of known HeV V binding partners now totals seven^9–13^. Based on our study and mapping studies conducted with HeV V, or the closely related NiV V, at least four of those partners map to the largely intrinsically disordered region of V spanning residues 1–405, which for unknown reasons is significantly larger in the *Henipaviruses*. Specifically, the binding sites of exportin-1, PLK1, STAT1 and STAT2 are clustered within residues 101–236^13,21^ (Fig. 7b). Thus, this intrinsically disordered 135-residue region that is shared in P/V/W can clearly adapt to bind to structurally diverse partners, representing a significant protein interaction hub^49,50^. It is therefore possible that drugs targeting this region could prevent HeV P/V/W interactions with multiple host factors and thereby very potently block suppression of the host antiviral response. Notably however, intrinsically disordered regions are challenging targets, and consequently such drugs are still in their infancy.

The nuclear role of HeV V is likely to be particularly pertinent to HeV pathogenesis as Cedar virus, which is also a *Henipavirus*, is hypothesized to be non-pathogenic in numerous animal models as it lacks the V protein. Accordingly, Cedar virus infection produces a more pronounced IFN-β response in human cells compared to HeV infection^51^. Thus, HeV V nucleocytoplasmic trafficking likely plays a role in antagonizing the IFN-I response and therefore represents a new opportunity to design novel anti-HeV therapeutics. Indeed, our study confirms that the nuclear import inhibitor ivermectin, as well as GSP, can block HeV infection in mammalian cells presumably by inhibiting V nuclear import by reducing recognition by importin α2/β1, (and/or potentially causing the formation of large dysfunctional complexes in the case of GSP). Significantly, ivermectin is already in use as a broad-spectrum anti-parasitic agent for animals, such as horses (Ivomec®), and is also approved by the U.S. Food and Drug Administration (FDA) for human parasitic infections (Stromectol®). Ivermectin thus appears an exciting possibility as a non-toxic FDA-approved agent to treat HeV infections in humans where they are usually fatal, as well as in horses in order to reduce the economic impact of HeV infection on the multi-million dollar equine industry.

That agents specifically targeting viral protein nuclear trafficking can be potent antiviral agents has already been shown for dengue virus^52^, and may also apply to HeV as implicated by our study here. Even more exciting, however, may be the possibility of devising agents that target a single region, or protein interaction hub, to prevent binding to multiple host factors. Flexible proteins present formidable challenges for drug design, however, our work provides a promising platform for the characterization of binding partners and disorder-to-order transitions that will facilitate future therapeutic strategies.

## Methods

### Cell culture

Vero, HEK293T and HeLa cell lines were routinely cultured at 37 °C with 5% CO_2_ in either Dulbecco’s Modified Eagles Media (Vero, HEK293T), or Eagle’s Minimal Essential Media (HeLa), supplemented with 10% foetal bovine serum and 2 mM L-glutamine.

### Antibodies

Antibodies to GFP (Roche Applied Science), actin (Abcam), importin α1 and exportin-1 (BD Biosciences), importin α3 (Abcam), and tubulin, FLAG and HA (Cell Signaling Technology) were from the indicated sources.

### HeV V expression constructs

The full-length HeV V gene (GenBank accession no. NC001906) from a HeV horse isolate (isolate Horse/Australia/Hendra/1994)^53^ was synthesized by GenScript® and provided in plasmid pUC57, and subsequently recloned into pLIC-His_6_-MBP, immediately downstream of a TEV protease cleavage site^54^. V truncations (constructs V_1-405_ and V_51-457_) as well as the NES double mutant (V_L174A/L177A_), were created by recloning the appropriate sequence into pLIC-His_6_-MBP or site-directed mutagenesis of the pLIC-His_6_-MBP-V construct, respectively.

For mammalian expression of P, V and W a HeV P gene cDNA template was used. To generate DNA corresponding to edited RNA encoding V and W for cloning into pEGFP-C1 (Clontech), additional G nucleotides were introduced into the RNA editing site of HeV P cDNA using overlap PCR mutagenesis. Non-mutated (+0 G) and mutated (+1 G and +2 G) P genes were inserted in-frame C-terminal to GFP using the *Bgl*II/*Bam*HI restriction sites of the multiple cloning site of the pEGFP-C1 plasmid. Two residues in the protein sequence of pEGFP-C1 V differ from that of pUC57 V (D118G and R217G). The pEGFP-C1 HeV V_L174A/L177A_ double mutant was created using overlap PCR mutagenesis of pEGFP-C1 HeV V. Plasmid pEGFP-C1, containing the NLS from SV40 large tumour antigen (T-Ag) (residues 111–135) has been described^55^. For coimmunoprecipitation assays, full-length HeV V in the pUC57 vector and full-length HeV V_L174A/L177A_ in the pEGFP-C1 vector were subcloned into the pcDNA vector downstream of a HA-tag using *Bam*HI/*Hind*III sites and *Bam*HI/*Xba*I sites, respectively.

### Expression and purification of HeV V

*E. coli* BL21(DE3) cells carrying plasmid pLIC-His_6_-MBP-V were grown in flasks containing 1 L Luria-Bertani broth (100 μg/ml ampicillin) at 37 °C with continuous shaking (200 rpm) until the culture reached OD_600_ ~ 0.6. The flasks were then incubated at 16 °C for 1 h before induction of protein expression with 1 mM isopropyl β-D-1-thiogalactopyranoside (IPTG). The cultures were then incubated overnight at 16 °C with continuous shaking before cells were harvested by centrifugation at 5000 rpm for 20 min.

The HeV V cell pellet was resuspended in Buffer I (20 mM Tris pH 8, 500 mM NaCl, 10% (v/v) glycerol, 20 mM imidazole, 5 mM β-mercaptoethanol) and 1 x protease inhibitor tablets (Roche mini-tabs EDTA-free) and the cells lysed by sonication using an MSE Soniprep with an 18 mm diameter probe at a power output of 10 μm (30 secs on, 60 secs off) for 20 min. Cellular debris were pelleted by centrifugation (18500 rpm, 1 h) and the soluble fraction was added to Ni-NTA agarose (Qiagen) equilibrated with buffer I, before incubation at 4 °C with inversion for 2 h. Resin was then washed with 4 column volumes of Buffer I to remove unbound proteins before elution with Buffer II (20 mM Tris pH 8.0, 500 mM NaCl, 10% (v/v) glycerol, 250 mM imidazole). Eluted protein was then concentrated using a 10 kDa cut-off centricon (Millipore) to 2 ml, and His_6_-MBP-V was isolated by size-exclusion chromatography using a Superdex 200 16/60 column (GE Healthcare) equilibrated with Buffer III (20 mM Tris pH 8.0, 150 mM NaCl, 10% (v/v) glycerol, 1 mM TCEP). His_6_-MBP-V was then incubated with TEV protease at a 20:1 ratio for 2 h at 20 °C to remove the His_6_-MBP tag. Cleaved protein was then concentrated using a 10 kDa cut-off centricon (Millipore) to 2 ml, and HeV V was isolated by size-exclusion chromatography using a Superdex 200 16/60 column (GE Healthcare) equilibrated with Buffer III. The purity of full-length and truncated HeV V, as well as that of all other recombinant proteins was >95%, as assessed by SDS-PAGE stained with Coomassie blue (Supplementary Fig. 11).

### Importin expression constructs

For CD, AUC, SAXS and AlphaScreen experiments the high expression mouse homologue of human importin α1/β1 (importin α2/β1) was used. The bacterial expression construct encoding His_6_-importin α2 in a pET30a vector was described previously^56^. Sequences encoding full-length-importin α2 and full-length importin β1 (mouse) were cloned into pGEX-2T immediately downstream of sequence encoding a GST tag, and therefore the constructs code for GST-importin α2 and GST-importin β1.

### Expression and purification of importin α2/β1

For CD, AUC and SAXS experiments all proteins were expressed separately in *E. coli* BL21(DE3) by induction with 0.5 mM IPTG for 18 h at 16 °C. Cells expressing GST-importin α2 and GST-importin β1 were lysed in 1 x PBS containing 5 mM dithiothreitol (DTT) and protease inhibitors, and purified by affinity chromatography using glutathione Sepharose 4B beads (GE Healthcare Life Sciences, PA). Thrombin was used to remove the GST-tag from importin α2 and importin β1, followed by size-exclusion chromatography with a Superdex 200 16/60 column (GE Healthcare) equilibrated with GF2 buffer (110 mM KCl, 5 mM NaHCO_3_, 5 mM MgCl_2_, 1 mM EGTA, 0.1 mM CaCl_2_, 20 mM HEPES, pH 7.4). The importin α2/β1 heterodimer was preformed by incubating equimolar amounts of importin α2 and importin β1 in IB buffer (110 mM KCl, 5 mM NaHCO_3_, 5 mM MgCl_2_, 1 mM EGTA, 0.1 mM CaCl_2_, 20 mM HEPES, 1 mM DTT, pH 7.4) at 20 °C for 30 mins.

To meet the requirements of the AlphaScreen assays the His_6_-importin α2/GST-importin β1 heterodimer was used. His_6_-importin α2 was expressed and purified as described previously^57^, and the expression and purification of GST-importin β1 was as described above, except the GST tag was not removed.

### Exportin-1 and Ran expression constructs

For AlphaScreen studies DNA encoding exportin-1 was ligated into pGEX-6P immediately downstream of sequence encoding a PreScission protease cleavable N-terminal GST-tag. Subsequently, a cassette encoding a TEV protease cleavable C-terminal His_6_-tag was ligated immediately downstream of the exportin-1 sequence to create a GST-exportin-1-His_6_ expression construct. For CD, AUC and SAXS experiments a GST-exportin-1 expression construct was generated by subcloning the DNA encoding GST-exportin-1 from pGEX-6P into pCOLD IV using the *Nde*I*/Kpn*I restriction sites. The pGEX-6P vector encoding GST-Ran was described previously^58^.

### Expression and purification of exportin-1 and Ran

For CD, AUC and SAXS experiments all proteins were expressed separately in *E. coli* BL21(DE3) by induction with 0.5 mM IPTG for 18 h at 16 °C. Cells expressing GST-exportin-1 or GST-Ran were lysed in 1 x PBS containing 5 mM dithiothreitol (DTT) and protease inhibitors, and purified by affinity chromatography using glutathione Sepharose 4B beads (GE Healthcare Life Sciences, PA). PreScission protease was used to cleave the GST tags off exportin-1 and Ran, followed by size-exclusion chromatography with a Superdex 200 16/60 column (GE Healthcare) equilibrated with GF1 buffer (20 mM Tris pH 7.5, 100 mM NaCl, 5 mM MgOAc, and 2 mM DTT). Subsequently 1 mM GTP (Sigma) was added to Ran and the Ran-GTP complex was further purified on a Superdex 200 16/60 column. The exportin-1/RanGTP complex was preformed by incubating equimolar amounts of exportin-1 and Ran-GTP in GF1 Buffer at 20 °C for 30 mins.

For AlphaScreen assays GST-exportin-1-His_6_ was expressed in *E.coli* BL21(pRep4) by induction with 1 mM IPTG for 18 h at 23 °C. Cells were lysed in Native Buffer (50 mM Tris, 500 mM NaCl) containing lysozyme and protease inhibitors and purified by affinity chromatography using glutathione Sepharose 4B beads (GE Healthcare) with 10 mM glutathione followed by dialysis and protein concentration. Subsequently 1 mM GTPγS (Sigma) was added to Ran and the Ran-GTPγS complex further purified on a Superdex 200 16/60 column. The exportin-1/Ran-GTPγS complex was preformed by incubating equimolar amounts of exportin-1 and Ran-GTPγS in Native Buffer at 20 °C for 30 mins.

### DNA and siRNA transfections

Plasmid transfections were performed using FuGENE HD (Promega) or Lipofectamine 2000 (Invitrogen) transfection reagents according to the manufacturer’s instructions. For siRNA knockdown of expression of specific importins or exportins, HeLa cells were transfected with SMARTpool ON-TARGET plus siRNA (Dharmacon, 25 nM final concentration) targeting *KPNA2* (importin α1), *KPNA4* (importin α3), *XPO1* (exportin-1), or scrambled (Scr) siRNA, using the Dharmafect transfection reagent #1 (Dharmacon) according to the manufacturer’s instructions. 48 h later cells were transfected with plasmids encoding GFP or GFP-fused paramyxovirus proteins using FuGENE HD for 18–24 h before live-cell imaging.

After imaging, cells were lysed in 1 x lysis buffer (Invitrogen) for western analysis. Image Studio Lite software v4.0.21 (Li-Cor) was used for densitometric analysis. The signal for each band was calculated, with subtraction of the median value of pixels in the surrounding background area. The background-corrected signal intensity for the band of interest (importin α1, importin α3 or exportin-1) was then normalized to the background-corrected signal intensity of the corresponding actin control band. Normalized expression levels of importin α1, importin α3 and exportin-1 in cells transfected with siRNA targeting *KPNA2*, *KPNA4* and *XPO1*, respectively, were then calculated as a percentage relative to their expression in cells transfected with Scr siRNA.

### Leptomycin B (LMB), ivermectin and Gossypol (GSP) treatments

For inhibition of exportin-1-dependent nuclear export in transfected cells, Vero cells were treated for 3.5 h with 10 nM LMB, before live-cell imaging. To assess the effects on HeV infection, cells were either pretreated for 3 h with different concentrations of ivermectin, or for 1 h with 10 μM GSP, prior to medium change and infection, or treated with 100 nM LMB at the time of infection with a wild-type HeV (multiplicity of infection (MOI) 0.05). Ivermectin and GSP at the appropriate concentrations were added back to the medium 2 h after infection. The toxicity of LMB and ivermectin was assessed by treating cells with the same concentration used for assessment of antiviral activity for 24 h, with media change at 8 h, and cell viability assessed using CellTiter-Glo® (Promega) system following the manufacturer’s recommendations.

### Confocal Laser Scanning Microscopy (CLSM)

Live-cell CLSM was performed using an Olympus Fluoview (FV) 1000 inverted microscope or Nikon Eclipse C1 inverted microscope with a 37 °C heated chamber. Images were taken using the Kalman scanning mode (minimal bleaching) using a 60 x water immersion lens. FIJI (v1.48q) software was used for analysis of digitized CLSM images to calculate the nuclear/cytoplasmic fluorescence ratio (Fn/c), after correction for background fluorescence, using an established approach for analysis at the single cell level^30,31,33,52,55,59,60^. Briefly, fluorescence in the linear range was measured in the nucleus and cytoplasm in each cell, and expressed as a ratio (corrected for background fluorescence). Single cell values were averaged for a minimum of 30 cells per sample/condition to correct for variations in expression levels.

### Immunoprecipitation

For importin α1 coimmunoprecipitation assays, transfected HEK293T cells were lysed with GFP-Trap lysis buffer (10 mM Tris pH 7.5, 150 mM NaCl, 0.5 mM EDTA, 0.5% (v/v) Nonidet P-40) containing 1 x cOmplete EDTA-free protease inhibitor cocktail tablet (Roche). After clarification cell extracts were incubated with GFP-Trap® (1 h; ChromoTek). Beads were washed with lysis buffer 5 times and protein complexes were eluted using 2 x SDS-PAGE loading buffer. Total cell lysate (input) and immunoprecipitate (IP) were separated by 10% SDS-PAGE for western transfer and immunoblotting.

For exportin-1 coimmunoprecipitation assays, transfected HEK293T cells were lysed with lysis buffer (20 mM Tris pH 7.5, 150 mM NaCl, 0.5% (v/v) Nonidet P-40), supplemented with 10 mM NaF, 1 mM PMSF, 1 mM Na_3_VO and 1 x cOmplete EDTA-free protease inhibitor cocktail tablet. Clarified lysates were incubated for 4 h with Protein G-coupled Dynabeads® magnetic resin (ThermoFisher Scientific #10003D) conjugated with mouse anti-exportin-1 antibody (BD Biosciences). Antibody-protein complexes were washed 3 times with lysis buffer without additives, then eluted using 2 x SDS-PAGE loading buffer, separated using SDS-PAGE and visualized by immunoblot.

### Circular dichroism spectroscopy

Circular dichroism (CD) spectra of HeV V in 10 mM Tris, 150 mM NaCl, 0.75 mM TCEP, pH 8.0 were recorded using a Jasco J-815 CD spectrometer. Spectra were recorded from 190 to 250 nm in a 1 mm quartz cuvette at 20 °C. The α-helical content was derived from the ellipticity at 222 nm as described in^61^.

Mean ellipticity values per residue (*θ*) were calculated as *θ* = (3300 × *m* × Δ*A*)/(*lcn*), where *l* is the path length (0.1 cm), *n* is the number of residues, *m* is the molecular mass in Daltons, and *c* is the protein concentration in mg/ml. Protein concentrations of 0.2 mg/ml were used when recording individual spectra and 0.15–0.2 mg/ml used for spectra of protein mixtures; for mixtures, the signal was converted to mean ellipticity values per residue (*θ*_obs_) as in^25^ via *θ* = 3300 Δ*A*/{[(*c*_1_ *n*_1_)/*m*_1_) + (*c*_2_ *n*_2_/*m*_2_)]*l*}, where *l* represents path length (0.1 cm), *n*_1_ or *n*_2_ is number of residues, *m*_1_ or *m*_2_ is molecular mass in Daltons, and *c*_1_ or *c*_2_ is protein concentration expressed in mg/ml for each of the two proteins in the mixture. The theoretical average ellipticity values per residue (*θ*_calc_) without secondary structure rearrangement within the protein mixture were calculated as follows: *θ*_calc_ = [(*θ*_1_ *n*_1_) + (*θ*_2_ *n*_2_ *R*)]/(*n*_1_ + *n*_2_ *R*), where *θ*_1_ and *θ*_2_ correspond to the measured mean ellipticity values per residue for the proteins separately, *n*_1_ and *n*_2_ to the number of residues for each of the two proteins, and *R* to the excess molar ratio of protein 2.

### Analytical ultracentrifugation

Sedimentation velocity experiments were conducted in a Beckman Coulter Optima analytical ultracentrifuge at a temperature of 20 °C. 380 μl of sample and 400 μl of reference solution (10 mM Tris, 150 mM NaCl, 10% (v/v) glycerol, 0.75 mM TCEP, pH 8.0) were loaded into a conventional double sector quartz cell and mounted in a Beckman 4-hole An-60 Ti rotor. Protein complexes were formed by incubating equimolar concentrations at room temperature for 30 mins prior to centrifugation. For ivermectin and GSP experiments, 10 or 50 μM inhibitor was added prior to incubation. Samples were centrifuged at a rotor speed of 40,000 rpm and the data was collected continuously at a single wavelength (280, 235 or 232 nm). Solvent density (1.0373 g/ml at 20 °C) and viscosity (1.4164 cP at 20 °C), as well as estimates of the partial specific volume (0.7215 ml/g for HeV V at 20 °C) and hydration estimate (0.4698 g/g) were computed using the program SEDNTERP^62^. Sedimentation velocity data at multiple time points were fitted to a continuous size [*c*(*s*)] and continuous mass [*c*(*M*)] distribution models^63^ using the program SEDFIT.

### Size exclusion chromatography in-line with small-angle X-ray scattering (SEC*-*SAXS*)*

Small angle X-ray scattering data were collected at the Australian Synchrotron on the SAXS/WAXS beamline. The X-ray beam size at the sample was 250 μm horizontal, 150 μm vertical and data were collected using a Pilatus 1 M detector positioned 1600 mm from the sample, giving a *q* range of 0.006–0.4 Å^−1^. Protein samples alone were subjected to in-line size exclusion chromatography on a Superdex 200 5/150 GL gel-filtration column (GE Healthcare) with a bed volume of 3 ml. Protein complexes were separated on a Superdex 200 10/300 gel-filtration column (GE Healthcare) with a bed volume of 24 ml. 50 μl sample or 100 μl complexes at 8–10 mg/ml were injected and the fractionated sample flowed through a 1.5 mm quartz capillary with a co-flow system^64^ where it was exposed to the X-ray beam. Data were collected at 16 °C with 500 detector images of sequential 1 and 5 s exposures for the 5/150 and 100/300 columns respectively.

Radial averaging, background subtraction and image series analysis was performed using scatterBrain (software package developed at the Australian Synchrotron). Five sequential images were averaged to generate each SAXS data set before subsequent analysis using the ATSAS 2.5.0 software^65^. Guinier fits and Kratky plots were made using PRIMUS and *P*(*r*) distribution analysis was performed using GNOM.

### AlphaScreen assay

AlphaScreen binding assays were performed as previously in triplicate in white opaque 384 well plates in a final volume of 25 μl^18,30^. 2 μl of 30 nM biotinylated V protein diluted in PBS was added to each well. 20 μl of pre-dimerized GST-exportin-1-His_6_/Ran-GTPγS or importin α2/β1 (His_6_-importin α2 with GST-importin β1) diluted in PBS were then added in serial dilutions ranging from 100 to 0.1 nM and incubated for 30 min at room temperature. Where indicated, LMB or ivermectin were pre-incubated with exportin-1/RanGTPγS or importin α2/β1, respectively, for 15 min prior to addition to the wells. 2 μl of nickel-chelate acceptor beads diluted 1:10 in PBS/1.25% BSA was added per well and incubated for 90 min at room temperature, followed by the addition of 1 μl of streptavidin-coated donor beads (diluted 1:10 in PBS) per well. Plates were incubated for a further 2 h and then read on an EnSpire plate reader (Perkin Elmer). Data was analyzed using SigmaPlot software (v.11; Systat Software), and *K*_*d*_ or IC_50_ values determined by fitting 3-parameter sigmoidal curves or one-phase association curves, respectively, to the data.

### Viruses and HeV infection

The reference strain for wild-type HeV and recombinant HeV was a clinical isolate of HeV (Hendra virus/Australia/Horse/1994/Hendra)^53^. Infection with HeV (MOI 0.05) without or with (MOI 0.5) firefly luciferase reporter was performed as previously in Vero cells under high security PC4 conditions at the Australian Animal Health Laboratory^20^. Infectious virus titration was measured by TCID_50_ as previously^20^, with luciferase activity measured using the Bright-Glo™ Luciferase Assay System^66^.

## Electronic supplementary material

Supplementary Information
