# Supplementary material for: Recognition by host nuclear transport proteins drives disorder-to-order transition in Hendra virus V
Source: Sci Rep. 2018 Jan 10;8:358. doi: 10.1038/s41598-017-18742-8 (PMC5762688; doi:10.1038/s41598-017-18742-8)
Supplement: Supplementary file 1 — Supplementary Information [file 41598_2017_18742_MOESM1_ESM.pdf]

SUPPLEMENTARY INFORMATION

Title: Recognition by host nuclear transport proteins drives disorder-to-order transition in Hendra virus V

Authors: Sarah C. Atkinson[§1], Michelle D. Audsley[§1], Kim G. Lieu[1], Glenn A. Marsh[2], David R. Thomas[1], Steven M. Heaton[1], Jason J. Paxman[3], Kylie M. Wagstaff[1], Ashley M. Buckle[1], Gregory W. Moseley[1], David A. Jans[*1], Natalie A. Borg[*1]

Running Title: HeV V gains structure upon binding to nuclear transport proteins

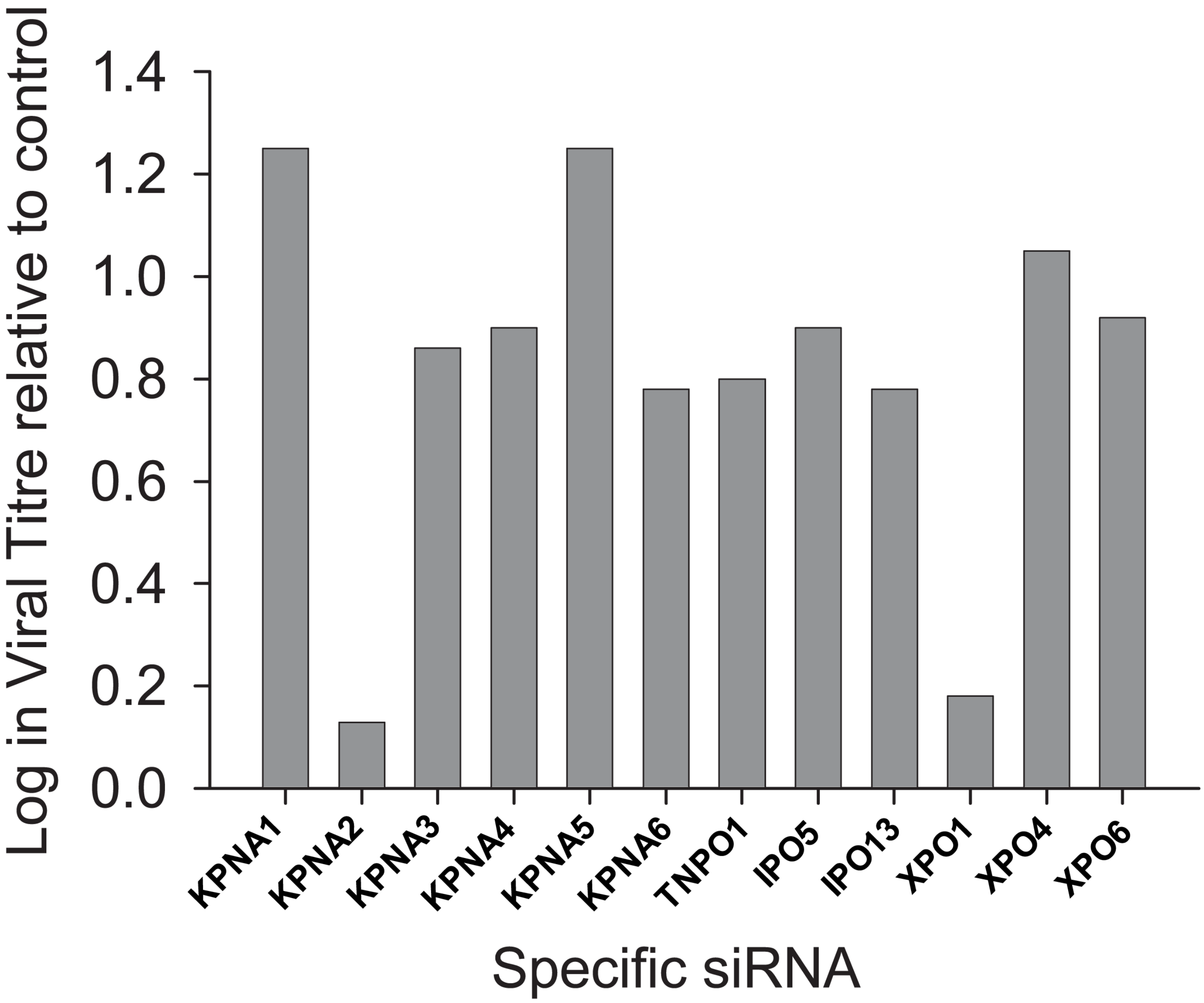

Supplementary Figure 1. *KPNA2* and *XPO1* are pivotal to HeV replication [20]. Genes were knocked down using siRNA prior to HeV infection and the impact on viral titre determined. It should be noted that siRNA treatments for *KPNB1* and *XPO5* were cytotoxic [20].

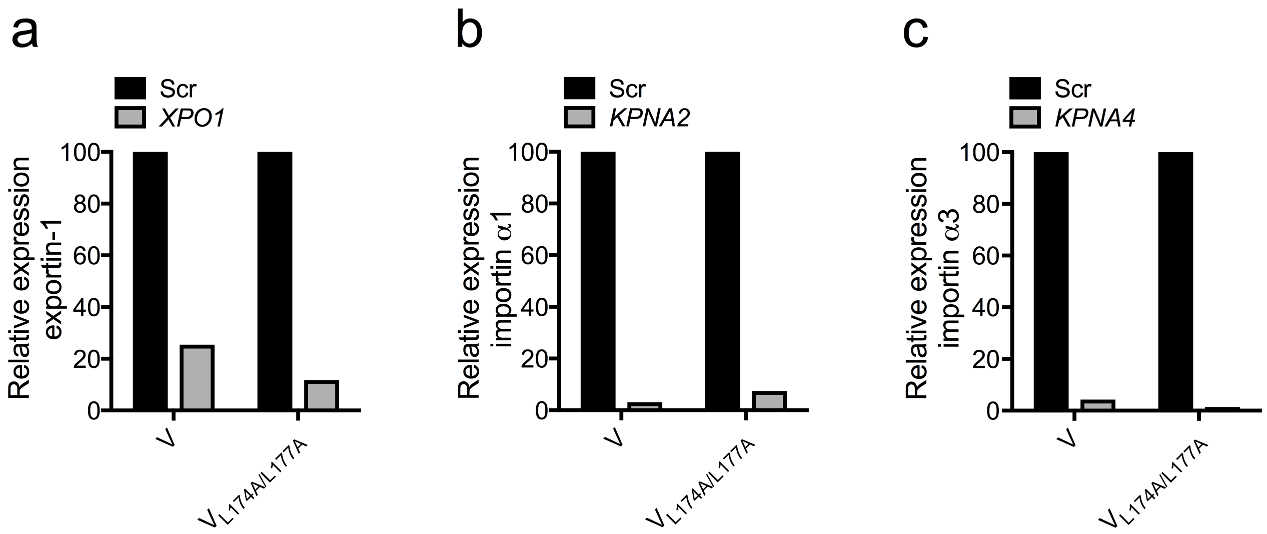

Supplementary Figure 2. Densitometry of western blots to estimate protein expression following siRNA treatment. Image Studio Lite software v4.0.21 (Li-Cor) was used to estimate expression of **(A)** exportin-1 from western blots in **Fig. 1e**, **(B)** importin α1 from western blots in **Fig. 2a**, and **(C)** importin α3 from western blots in **Fig. 2b**. For each sample, the expression level of exportin-1 or importins was normalized to actin expression. End values were calculated relative to values for the control (Scr) siRNA transfected samples.

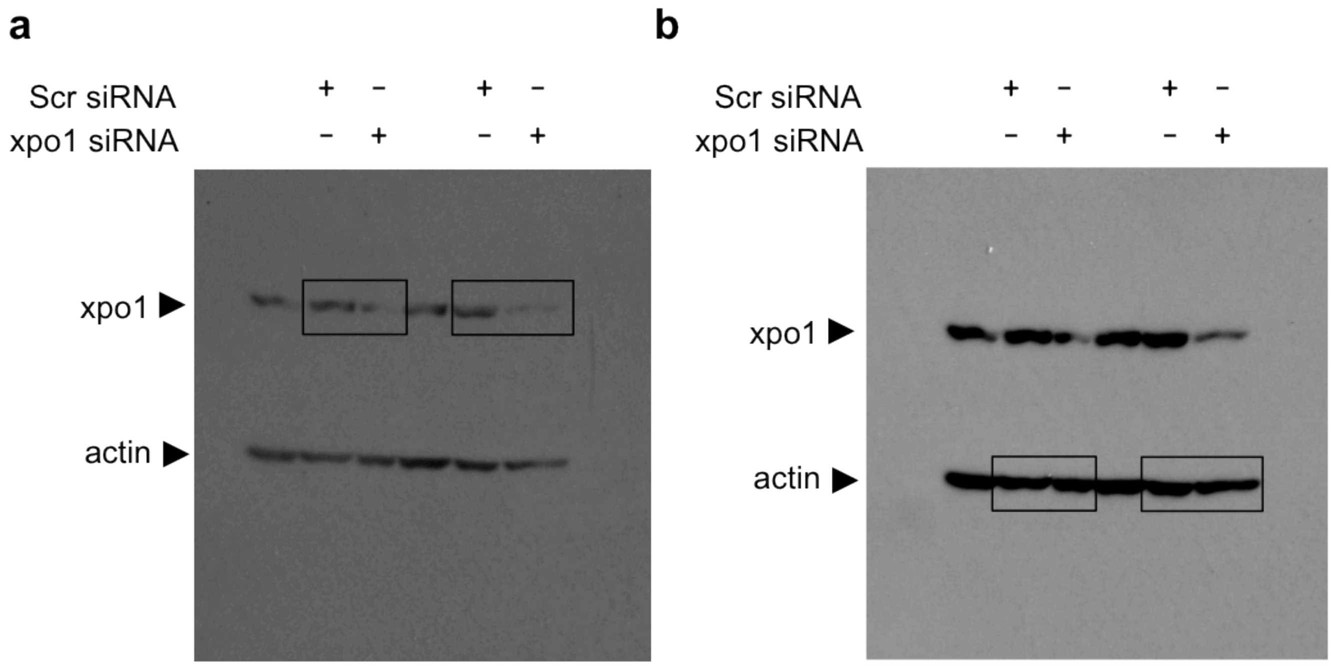

Supplementary Figure 3. Unprocessed western blot for xpo1 and control actin expression levels relating to Figure 1e. Black boxes indicate data used in **Fig. 1e**. **(B)** is a longer exposure of the X-ray film in (A). Description of the experiment as per the legend to **Fig. 1e.**

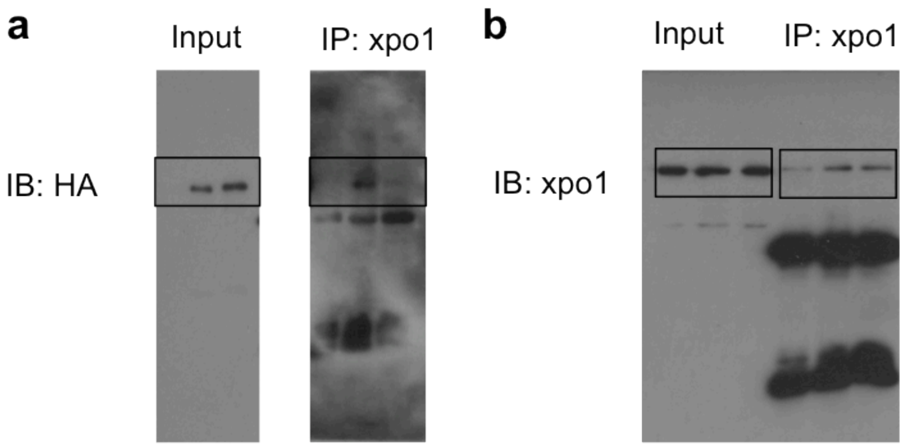

Supplementary Figure 4. Unprocessed western blot relating to Figure 1h. Black boxes indicate data used in **Fig. 1h**. Description of the experiment as per the legend to **Fig. 1h**.

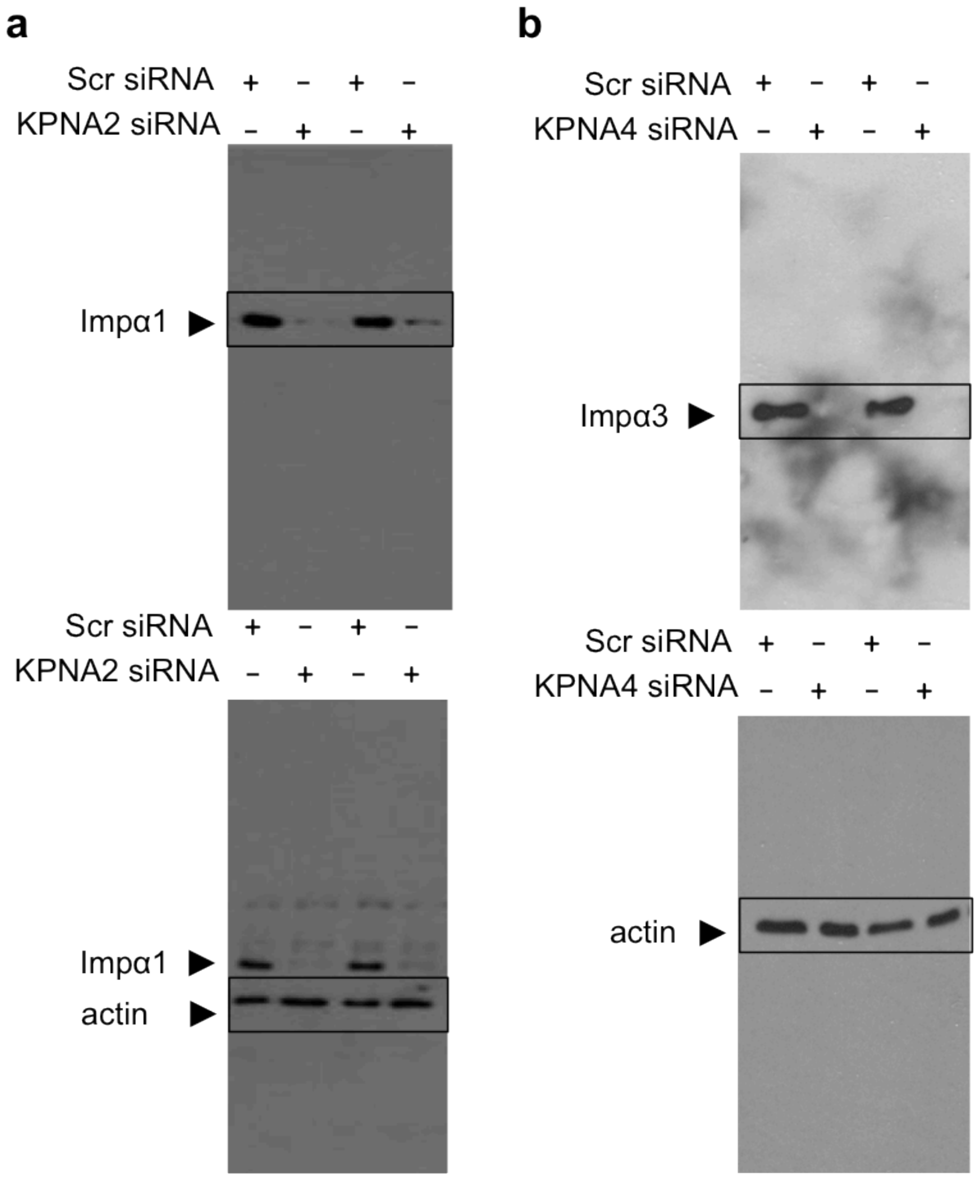

Supplementary Figure 5. Unprocessed western blot for importin α1, importin α3 and actin control expression levels relating to Figure 2b. Black boxes indicate data used in **Fig. 2b**. Description of the experiment as per the legend to **Fig. 2b**.

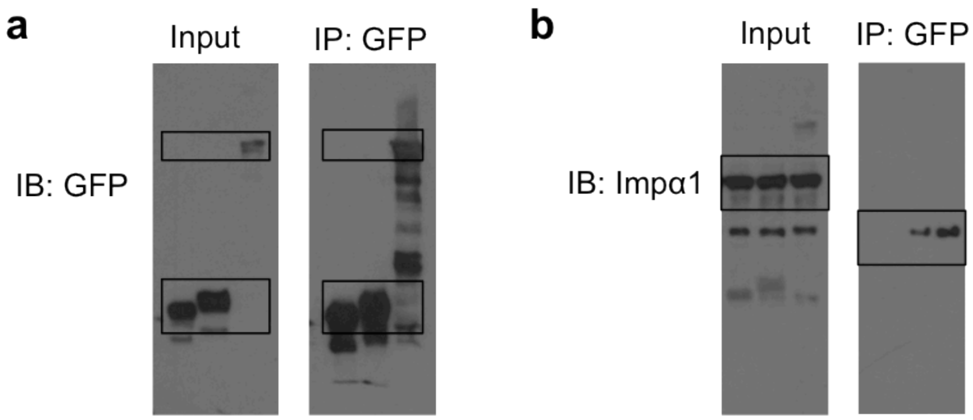

Supplementary Figure 6. Unprocessed western blot for GFP and importin α1 relating to Figure 2e. Black boxes indicate data used in **Fig. 2e**. Description of the experiment as per the legend to **Fig. 2e**.

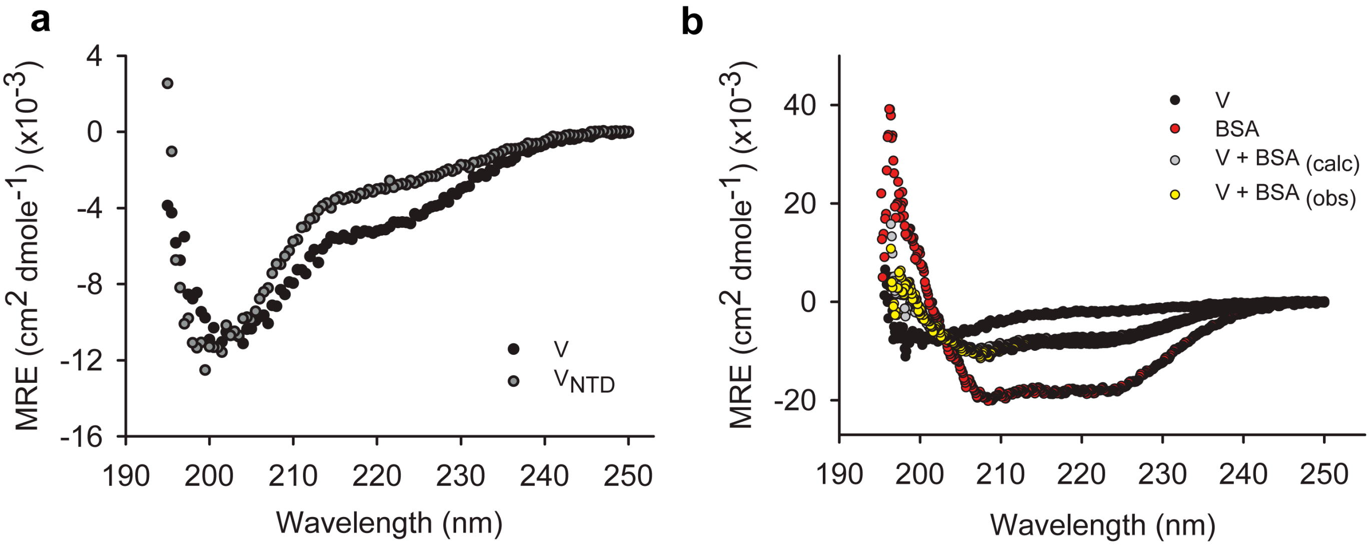

Supplementary Figure 7. N-terminal HeV V residues 1-405 are predominantly disordered. (A) CD spectra of full-length HeV V (*black*) and $V_{1\text{-}405}$ (*gray*). **(B)** CD spectra of V (*black*) and BSA (*red*), as well as equimolar mixtures of V:BSA (*yellow*) (obs). The theoretical average curves (calc) calculated by assuming that no structural variations occur (see Methods) are shown in *gray*.

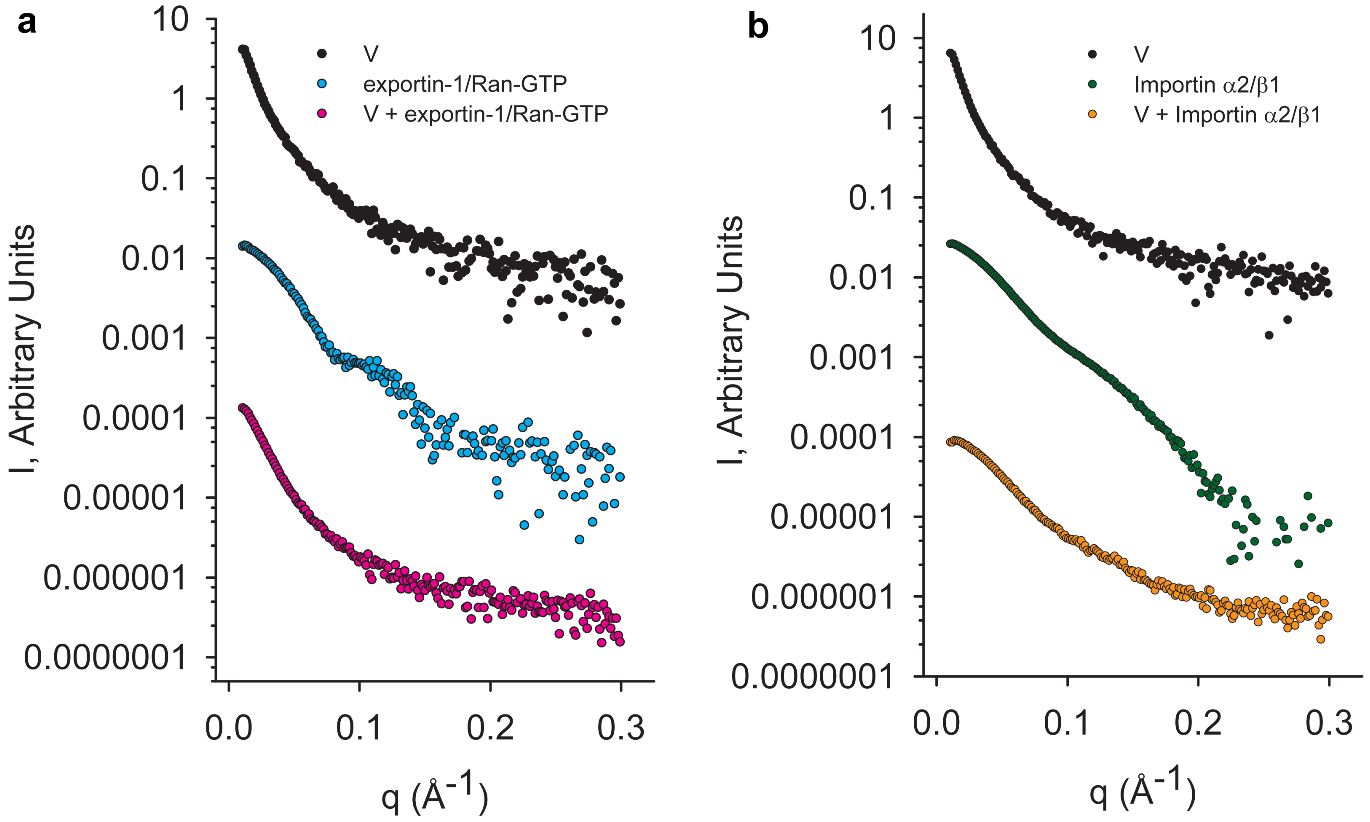

Supplementary Figure 8. High-quality SAXS scattering curves of HeV V alone and in complex. (A) Scattering curves for V, exportin-1 and the V:exportin-1/Ran-GTP complex at a 1:1 molar ratio of monomers. The V and complex curves were scaled by 100 and 0.001, respectively, to eliminate overlap. **(B)** SAXS cattering curves for V, importin α2/β1 and the V:importin α2/β1 complex at a 1:1 molar ratio of monomers. The V and complex curves were scaled by 100 and 0.001 to eliminate overlap.

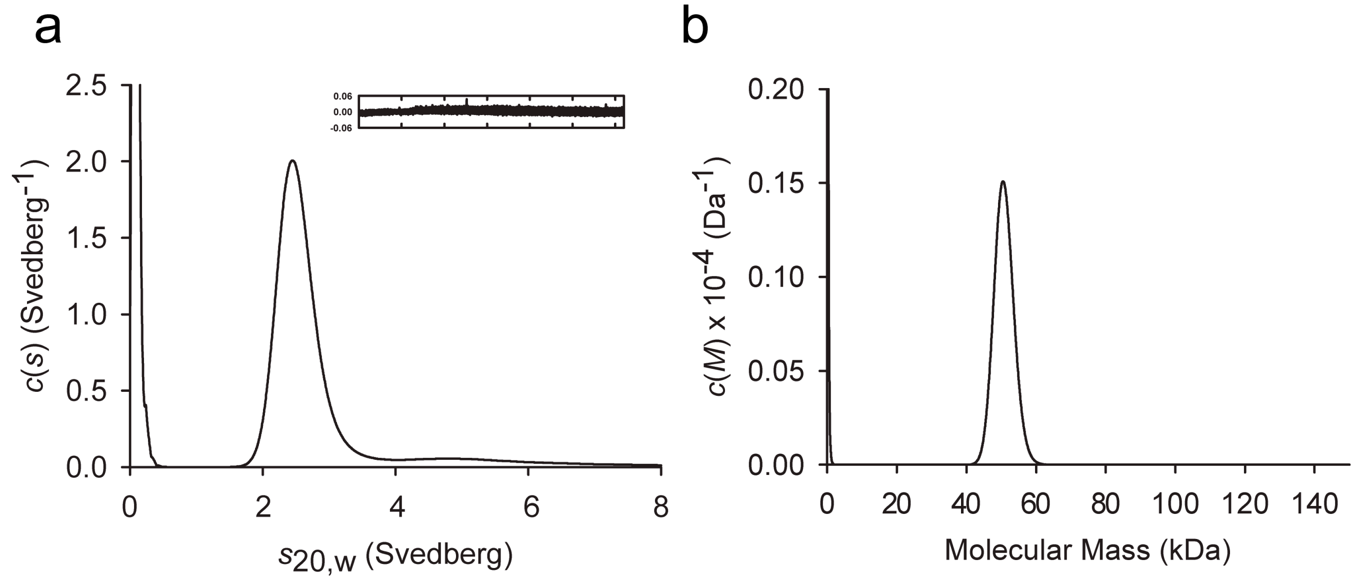

Supplementary Figure 9. **Sedimentation velocity experiments reveal monomeric full-length HeV V.** **(A)** Continuous size [*c(s)*] distribution is plotted as a function of size (Svedberg). Continuous size-distribution analysis was performed using the program SEDFIT [55] at a resolution of 200, with $S_{\min} = 0.1$, $S_{\max} = 20$ and at a confidence level (F-ratio) = 0.95. Residuals plotted as a function of radial position (cm) from the axis of rotation are shown above **(B)**. Continuous mass, *c(M)*, distribution of V plotted as a function of molecular mass (kDa).

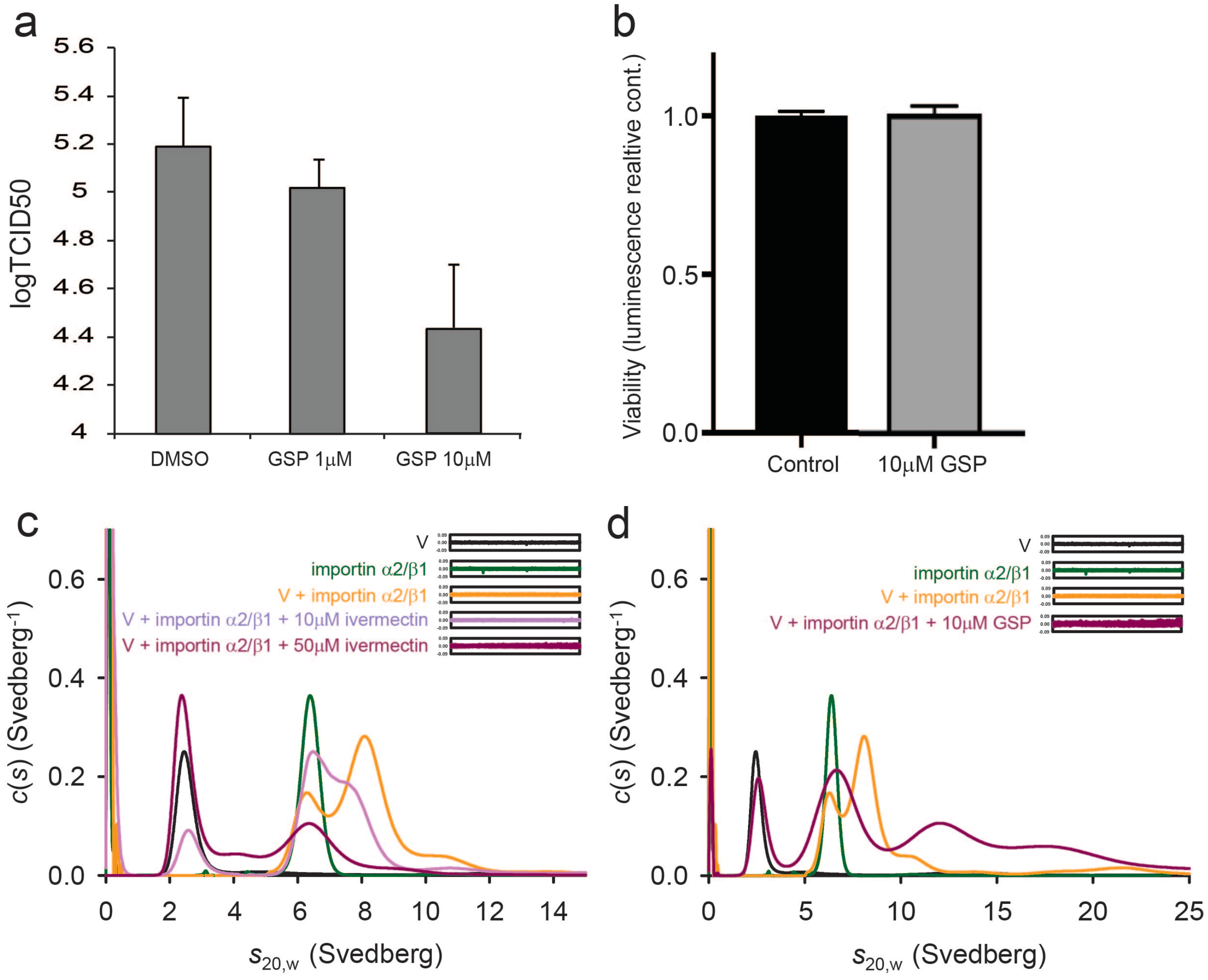

Supplementary Figure 10. Ivermectin and GSP inhibit HeV production and V:importin α2/β1 complex formation. (A) Vero cells were pretreated with the indicated concentrations of Gossypol (GSP) 1 h prior to infection with HeV, and GSP was maintained in the medium after infection. Production of infectious virus was measured by $TCID_{50}$ at 24 h post-infection (p.i.) as per the legend to **Fig. 6e**. Results represent the mean + SEM (n = 3) **(B)** Vero cells treated with inhibitors as per (A) were analyzed for cytotoxicity using CellTiter-Glo® (Promega) as per the legend to **Fig. 6g**. (**C,D**) Sedimentation velocity analytical ultracentrifugation experiments were performed to assess the effect of ivermectin **(C)** and GSP **(D)** on V:importin α2/β1 complex formation. The continuous sedimentation coefficient distribution [(*c*)*s*] was plotted as a function of $s_{20,w}$ for HeV V (black), importin α2/β1 (green), an equimolar V:importin α2/β1 mixture alone (orange) and in the presence of inhibitor (purple). Residual plots shown in insets.

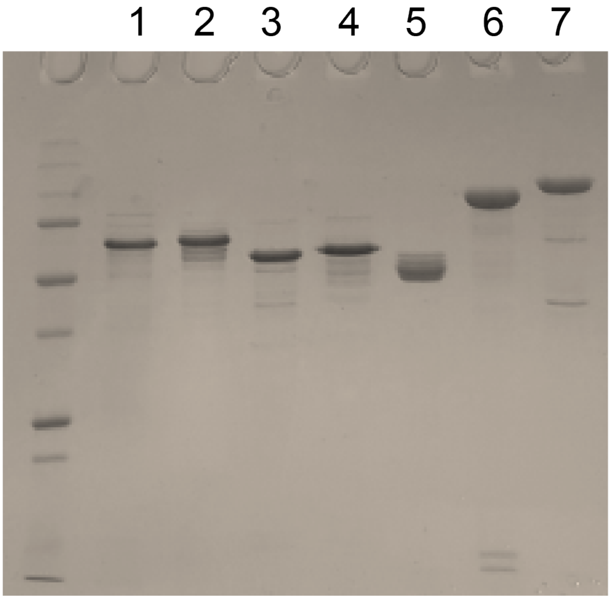

Supplementary Figure 11. **SDS-PAGE of recombinant purified proteins.** Coomassie blue-stained SDS-PAGE showing purified HeV V (lane 1), $V_{L174A/L177A}$ (lane 2), $V_{1\text{-}405}$ (lane 3), $V_{51\text{-}457}$ (lane 4), importin α2 (lane 5), importin β1 (lane 6) and exportin-1 (lane 7).

Supplementary Table 1: Hydrodynamic properties of recombinant proteins and their resultant complexes, related to Figure 3.

	M_r[a]	M[b]	$s_{20,w}$[c]	f/f_0[d]
V	50648	50316	2.5	3.2
importin α2	58072	50316	3.7	2.4
importin β1	98557	106358	5.8	2.1
importin α2/β1	156629	155055	6.4	2.7
V + importin α2/β1	207277	207043	8.1	3.3
exportin-1	123386	117954	4.9	1.7
Ran-GTP	24423	25682	2.2	1.3
exportin-1/Ran-GTP	147809	13003	5.1	1.7
V + exportin-1/Ran-GTP	198457	190516	6.7	3.2
$V_{1\text{-}405}$	44451	43809	2.7	3.3
$V_{51\text{-}457}$	44738	41709	2.4	3.2
$V_{L174A/L177A}$	50564	52552	2.4	3.3

[a] Relative molecular weight calculated from the amino acid sequence

[b] Molar mass determined from the ordinate maximum of c(M) distribution best fits (data not shown)

[c] Standardized sedimentation coefficient taken from the ordinate maximum of the c(s) distribution best fits (**Fig. 3**)

[d] Frictional coefficient calculated from $s_{20,w}$ using the $\bar{v}$ method employing SEDNTERP
